# Review on MXenes-Based Electrocatalysts for High-Energy-Density Lithium–Sulfur Batteries

**DOI:** 10.1007/s40820-025-01726-z

**Published:** 2025-04-10

**Authors:** Xintao Zuo, Yanhui Qiu, Mengmeng Zhen, Dapeng Liu, Yu Zhang

**Affiliations:** 1 Hangzhou International Innovation Institute, Beihang University, Hangzhou, 311115 People’s Republic of China; 2 School of Chemistry, Beihang University, Beijing, 100191 People’s Republic of China; 3 School of Energy and Environmental Engineering, Hebei University of Technology, Tianjin, 300071 People’s Republic of China

**Keywords:** High-sulfur loading, Lean electrolyte, Shuttle effect, MXenes, Lithium–sulfur batteries

## Abstract

Lithium–sulfur batteries (LSBs) hold significant promise as advanced energy storage systems due to their high energy density, low cost, and environmental advantages. However, despite recent advancements, their practical energy density still falls short of the levels required for commercial viability. The energy density is critically dependent on both sulfur loading and the amount of electrolyte used. High-sulfur loading coupled with lean electrolyte conditions presents several challenges, including the insulating nature of sulfur and Li_2_S, insufficient electrolyte absorption, degradation of the cathode structure, severe lithium polysulfide shuttling, slow redox reaction kinetics, and instability of the Li metal anode. MXenes-based materials, with their metallic conductivity, large polar surfaces, and abundant active sites, have been identified as promising electrocatalysts to improve the redox reactions in LSBs. This review focuses on the significance and challenges associated with high-sulfur loading and lean electrolytes in LSBs, highlighting recent advancements in MXenes-based electrocatalysts aimed at optimizing sulfur cathodes and lithium anodes. It provides a comprehensive discussion on MXenes as both active materials and substrates in LSBs, with the goal of enhancing understanding of the regulatory mechanisms that govern sulfur conversion reactions and lithium plating/stripping behavior. Finally, the review explores future opportunities for MXenes-based electrocatalysts, paving the way for the practical application of LSBs.

## Highlights

- The significance and challenges associated with high-sulfur loading and lean electrolytes in lithium–sulfur batteries are comprehensively reviewed.
- Catalytic properties of MXenes-based electrocatalysts are optimized via d-band center tuning, internal electric field constructing, single-atom seeding, and cocktail effects introducing.
- The structure–activity relationships between MXenes-based electrocatalysts and lithium–sulfur battery performances are comprehensively summarized.

## Graphical abstract

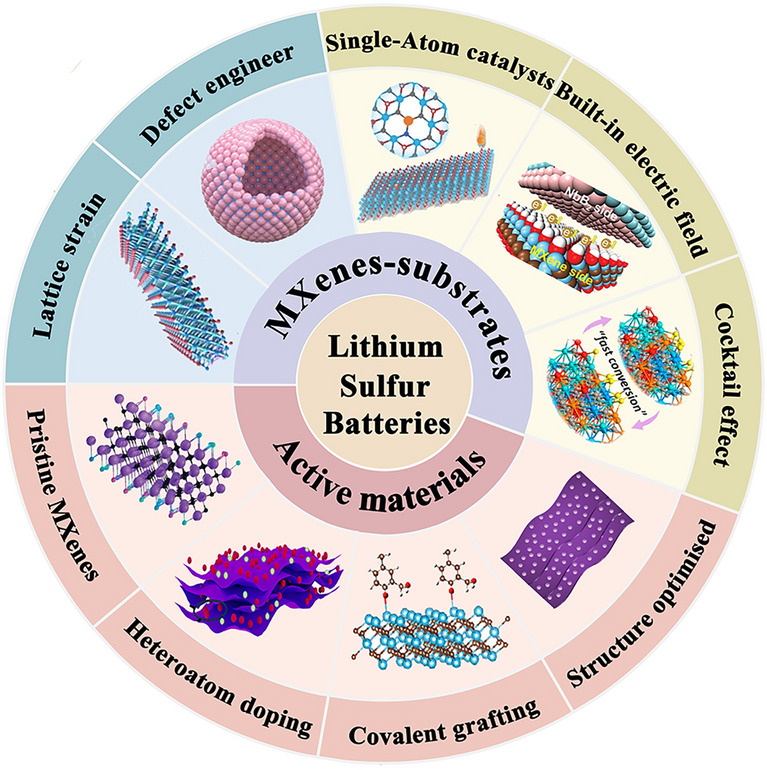

## Introduction

The increasing dependence on fossil fuels causes large-scale emissions of greenhouse gases and harmful pollutants and thus exacerbates the global energy crisis and intensifies environmental issues [1, 2]. Under this background, renewable energy sources like solar, wind, and biomass have garnered significant attention for their inexhaustibility and environmental sustainability in the past decades [3, 4]. However, the inherent intermittency and instability of these energy sources limit their practical applications, creating an urgent need for advanced energy storage systems capable of converting and storing renewable energy for stable and continuous power supply [5]. Among the available systems, rechargeable batteries have been widely applied in powering mobile devices, smart grids, and electric vehicles [6]. Lithium-ion batteries (LIBs) dominate the market for portable electronics, yet their energy density is approaching its theoretical maximum, making them inadequate for the growing demands of next-generation power applications [7]. Moreover, commercially available LIBs, with a relatively low energy density of no more than 300 Wh kg^−1^, cannot meet the demand for longer endurance, such as more than 300 miles for electric vehicles [8], which require an energy density of 500 Wh kg^−1^.

**Fig. 1:**
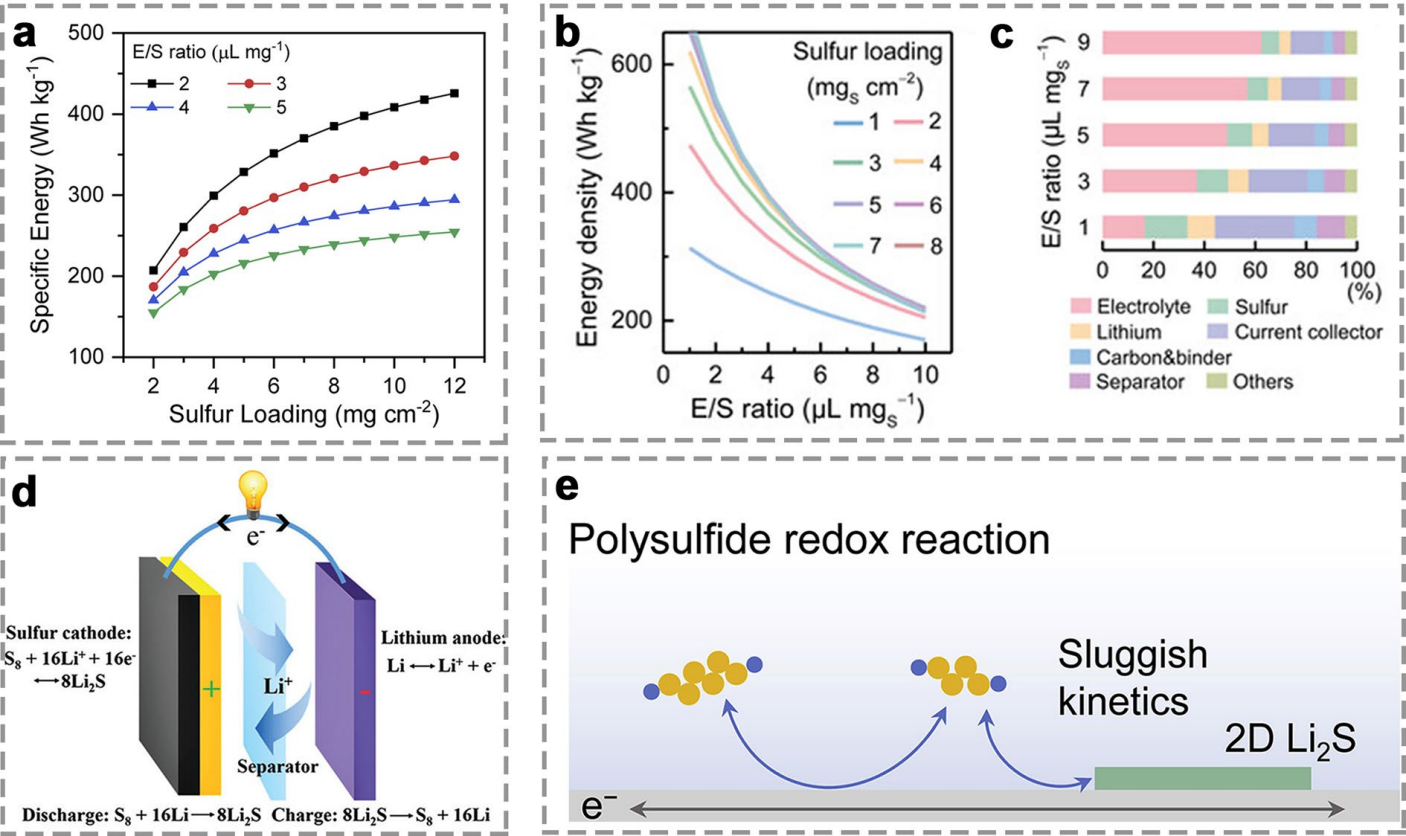
**a** Plots of sulfur loading versus specific energy at different E/S ratios [20]. Copyright 2020, Elsevier. **b** Effect of E/S ratios on energy density of LSBs, and **c** mass ratios of various components at different E/S ratios [21]. Copyright 2019, Wiley–VCH. **d** Schematic of LSB electrochemistry [24]. Copyright 2016, Royal Society of Chemistry. **e** Schematic of sulfur redox reactions for LSBs [25]. Copyright 2020, Elsevier

Compared to LIBs, lithium–sulfur batteries (LSBs), involving the multi-electron redox conversion mechanism, have emerged as a promising alternative, which can offer superior energy storage capabilities [9–12]. By utilizing elemental sulfur as cathode and Li metal as anode, LSBs can theoretically deliver an energy density as high as 2600 Wh kg^−1^, approximately six times higher than that of conventional LIBs [13–15]. Energy density relying on sulfur loading is a key metric for assessing the practical performance of batteries. In general, low-sulfur loading and excessive electrolyte usage in LSBs enable significantly improved specific capacity (> 1000 mAh g^−1^), rate performance (> 40C), and cycling stability (> 1500 cycles) [16–18]. However, low-sulfur loading and high electrolyte-to-sulfur (E/S) ratios will greatly reduce energy density and increase electrolyte costs, limiting commercial viability [19]. LSBs usually operate at an average voltage of 2.15 V (lower than 3.60 V of typical LIBs), so an areal capacity of 4.0–8.0 mAh cm^−2^ seems to be required to compete effectively [19]. Research has shown that a sulfur loading less than 2.0 mg cm^−2^ cannot achieve the energy density of 500 Wh kg^−1^ under any E/S ratio (Fig. 1a, b) [20, 21]. Moreover, if the E/S ratio exceeds 10 µL mg^−1^, the electrolyte should account for more than 50% of the total weight (Fig. 1c) [21]. However, when the E/S ratio is reduced from 5.0 to 2.0 µL mg^−1^, it can boost the specific energy by over 50%. That means achieving an energy density above 500 Wh kg^−1^ requires high-sulfur loadings (> 5.0 mg cm^−2^) as well as low E/S ratios (< 5.0 µL mg^−1^) [22]. In the past decade, significant improvements have been achieved especially in enhancing specific capacity, sulfur utilization, and cycling life of LSBs; however, they are generally realized under low-sulfur loading (< 2.0 mg cm^−2^) and excessive electrolyte usage, with an electrolyte-to-sulfur (E/S) ratio exceeding 15.0 μL mg^−1^ [23]. Hence, significant challenges remain in developing advanced materials capable of operating under high-sulfur loading and lean electrolyte conditions to meet the practical application and commercialization requirements.

**Fig. 2:**
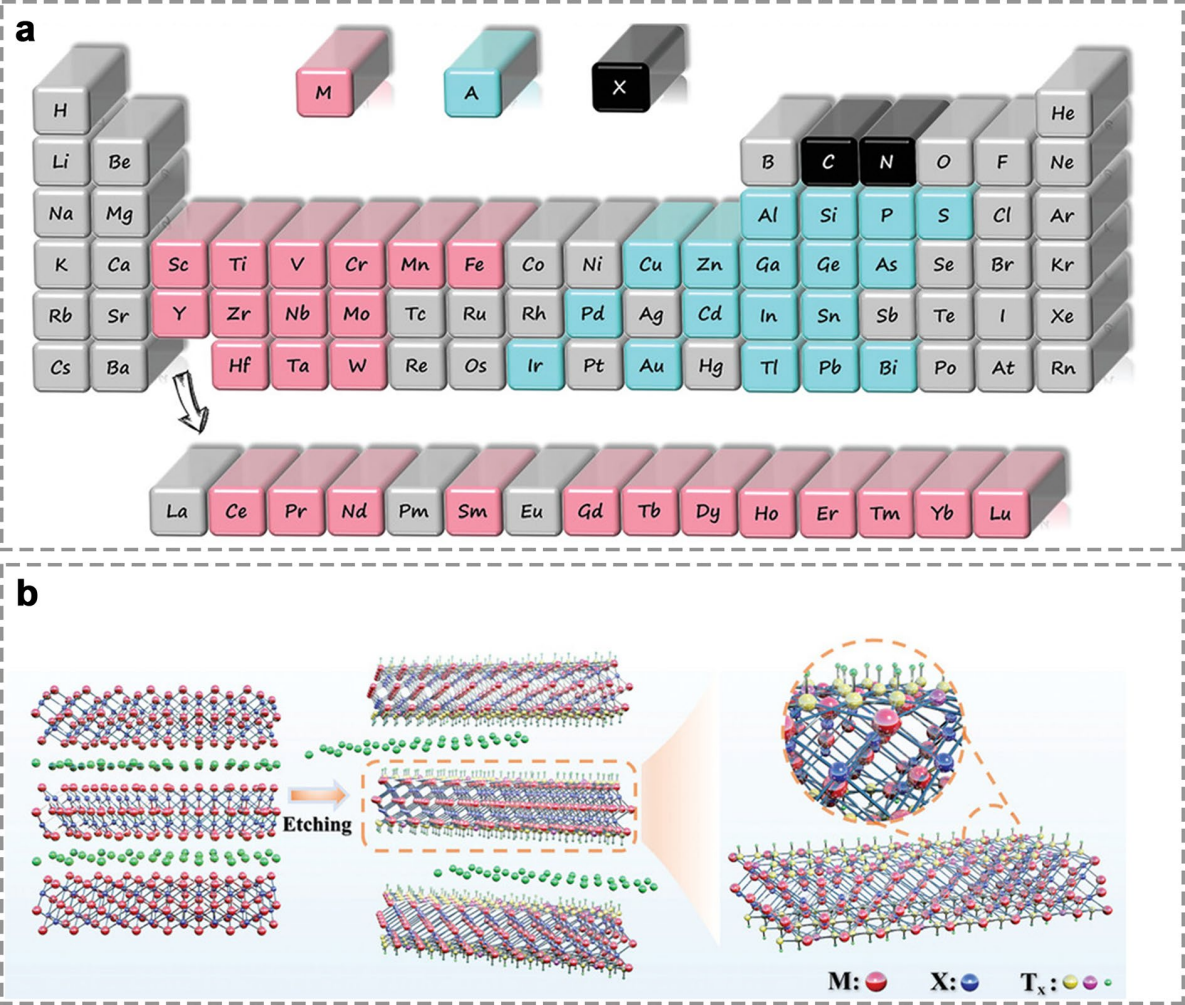
**a** The brief representation of MAX [24]. Copyright 2021, WILEY–VCH. **b** The schematic diagram of preparation process of MXenes [28]. Copyright 2024, WILEY–VCH

As a class of two-dimensional (2D) materials, MXenes have shown significant potential in energy storage applications [26, 27]. MXenes are synthesized by selectively removing A layers from MAX phases [28], which are generally represented by the formula of M_n+1_X_n_T_x_. In this formula, M denotes a transition metal (e.g., Ti, V, Zr, Nb), A represents a group IIIA or IVA element (e.g., Al, Ga, Si), X corresponds to carbon (C) or nitrogen (N), and T_x_ refers to surface terminations like − O, − OH, − F, and − Cl (Fig. 2a, b) [29]. Currently, over 150 MAX phases and more than 30 types of MXenes have been experimentally synthesized, with new variants continuously emerging [24, 25]. Unlike many other single-composition 2D materials, MXenes exhibit diverse compositions and possess unique properties, including metallic conductivity, large active surfaces, strong mechanical strength, and high surface area [26]. These characteristics make MXenes highly promising for applications in electrocatalysis, electromagnetic shielding, energy storage, and biomedicine [27].

Recently, novel MXenes-based electrocatalysts have emerged in large numbers for LSBs [30, 31]. As hosts for sulfur and Li, they provide suitable structures and abundant catalytic active sites that promote the conversion of high-concentration lithium polysulfide (LiPSs), facilitate the nucleation/decomposition of Li_2_S, and inhibit the growth of Li dendrites, thereby improving the practical energy density of LSBs [32, 33]. However, a comprehensive overview of MXenes-based electrocatalysts for high-energy–density LSBs is still lacking, which is essential to address the significant challenge posed by high-sulfur loading and lean electrolyte conditions. This review aims to explore recent advancements over the past five years, providing insights into novel MXenes-based electrocatalysts with high stability and excellent electrochemical performance. It also examines the structure–activity relationships of MXenes to reveal how they synergistically optimize the redox conversions of LiPSs and Li_2_S, and promote uniform Li deposition, ultimately bridging the gap between practical and ideal LSB systems. Finally, the review discusses the design principles for high-efficiency electrocatalysts, as well as theoretical calculations and in situ characterizations of catalytic mechanisms, making it highly relevant to researchers in the fields of chemistry, materials science, and energy storage.

## Electrochemical Reaction Mechanisms of LSBs

A typical LSB usually consists of sulfur cathode, separator, electrolyte, and Li metal anode, whose electrochemistry is based on the multi-electron reversible redox between S₈ molecules and Li metal [34–38], involving the sulfur reduction reaction (SRR) during discharge and sulfur evolution reaction (SER) during charge as shown in Fig. 1d [39–41].

### The SRR of Sulfur Cathode

S_8_ (solid) → LiPSs (liquid) (contributing ~ 25% theoretical discharge capacity)

1 $${\text{S}}_{{8}} + {\text{2Li}}^{ + } + {\text{2e}}^{ - } \to {\text{Li}}_{{2}} {\text{S}}_{{8}}$$

2 $${\text{3Li}}_{{2}} {\text{S}}_{{8}} + {\text{2Li}}^{ + } + {\text{2e}}^{ - } \to {\text{4Li}}_{{2}} {\text{S}}_{{6}}$$

3 $${\text{2Li}}_{{2}} {\text{S}}_{{6}} + {\text{2Li}}^{ + } + {\text{2e}}^{ - } \to {\text{3Li}}_{{2}} {\text{S}}_{{4}}$$

LiPSs (liquid) → Li_2_S (solid) (contributing ~ 75% theoretical discharge capacity)

4 $${\text{Li}}_{{2}} {\text{S}}_{{4}} + {\text{2Li}}^{ + } + {\text{2e}}^{ - } \to {\text{2Li}}_{{2}} {\text{S}}_{{2}}$$

5 $${\text{Li}}_{{2}} {\text{S}}_{{4}} + {\text{6Li}}^{ + } + {\text{2e}}^{ - } \to {\text{4Li}}_{{2}} {\text{S}}$$

6 $${\text{Li}}_{{2}} {\text{S}}_{{2}} + {\text{2Li}}^{ + } + {\text{2e}}^{ - } \to {\text{2Li}}_{{2}} {\text{S}}$$

During the initial stage of the discharge process, the S–S bonds in solid rhombic S_8_ are cleaved, and the resulting sulfur atoms combine with Li^+^ to form liquid Li_2_S_8_ at a voltage of ~ 2.4 V (Eq. (1)). This is followed by the reduction of Li_2_S_8_ to the lower-order polysulfide Li_2_S_4_ within the voltage range of 2.3 to 2.1 V (Eqs. (2) and (3)). This conversion occurs near thermodynamic equilibrium [42]. Subsequently, liquid Li_2_S_4_ is further reduced to solid Li_2_S_2_ or Li_2_S in the voltage range of 1.9 to 2.1 V, with both reduction processes occurring simultaneously (Eqs. (4) and (5)) [43]. Finally, the remaining Li_2_S_2_ converts to Li_2_S through a single-phase reaction (Eq. (6)). Notably, the reduction of Li_2_S_2_ to Li_2_S is typically considered the rate-determining step in sulfur chemistry due to the sluggish solid-to-solid conversion kinetics and high overpotentials (Fig. 1e) [44, 45].

### The SER of Sulfur Cathode

7 $${\text{8Li}}_{{2}} {\text{S}} \to {\text{S}}_{{8}} + {\text{16Li}}^{ + } + {\text{16e}}^{ - }$$

In the charging process, solid Li_2_S is first oxidized to LiPSs and undergoes delithiation through oxidation and disproportionation reactions [46, 47]. The formation and consumption of Li_2_S_6_, along with the deposition of insulating Li_2_S, slow down the conversion process, requiring higher overpotentials to drive the reaction [48]. Due to the electrochemical inertness of Li_2_S, additional activation energy is needed for its decomposition. Li_2_S requires extra activation energy for decomposition due to its electrochemical inertness [49]. The overall decomposition of Li_2_S occurs in two steps: first, a Li^+^ ion dissociates from the Li_2_S structure, and then the dissociated Li^+^ ion diffuses away from the LiS cluster [50]. The decomposition barriers for Li_2_S are considerably larger than the Li^+^ diffusion barriers, indicating that the breaking of the Li–S bond is the rate-limiting step in the process.

During SRR/SER, the insulating nature of sulfur and Li_2_S/Li_2_S_2_ greatly affects the electron transport and increase the internal resistance of battery, resulting in inefficient sulfur utilization [51, 52]. Due to intrinsic metallic conductivity, pristine MXenes as sulfur host or separator modifier can reduce the battery resistance [32, 33, 49, 50]; however, their limited porosity goes against high-sulfur loading. As sulfur loading increases and E/S ratio reduces, the cathode will suffer the severe LiPSs diffusion problem [53, 54]. Meanwhile, if the electrolytes contain high-concentration LiPSs, the deposition of Li_2_S becomes slow and it begins to uncontrollably accumulate, further aggravating the solid/liquid interface passivation [55, 56]. Despite pristine MXenes can steadily anchor soluble LiPSs through metal-S bonds [53–56], the irreversible restacking of MXene layers induced by van der Waals interactions and hydrogen bonding often decreases the exposure of active sites and the specific surface area, which causes serious performance degradation on capturing and catalyzing LiPSs [57]. To address this, MXenes can serve as substrates to support active components to, creating synergistic interfaces [30, 57], which in turn promote the catalytic conversion of LiPSs and ensure uniform Li_2_S deposition [58, 59].

As for anode, the uncontrolled growth of Li dendrites should be mainly responsible for the consequent potential risk of battery short circuit [60, 61]. Compared to LIBs, the operation of LSBs seems more complicated due to the direct contact of LiPSs with Li anode [62–64]. Pristine MXenes can be employed as Li host to inhibit Li dendrites, because their surface terminations provide abundant nucleation sites for guiding uniform deposition of Li^+^ [63]. Besides, the metallic conductivity and low Li^+^ diffusion energy barrier of MXenes do help accelerate electron/Li^+^ transport and further the electrochemical kinetics [60, 62]. Under lean electrolyte conditions, the wettability of both sulfur cathode and Li anode is very important for long cycles. However, some negatively charged surface terminations (e.g., -F or -Cl) are unfavorable for wetting, leading to incomplete contact between MXenes and electrolyte [65]. Also, there is a storage problem for pristine MXenes by virtue of their instability towards oxygen-rich environments [66].

## MXenes as Active Materials and Substrates for High Loading and Lean Electrolyte LSBs

### MXenes as Active Materials

#### Pristine MXenes

**Fig. 3:**
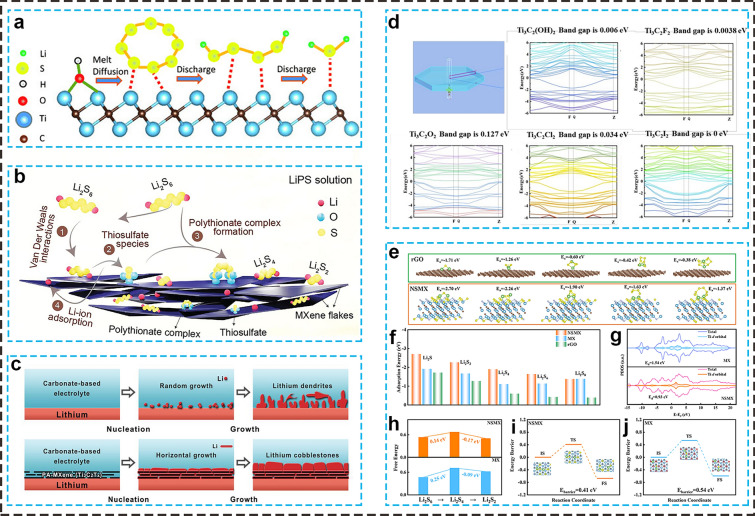
**a** Schematic illustration of LiPSs conversion process on Ti_2_CT_x_-MXenes surface [67]. Copyright 2015, WILEY–VCH. **b** Schematic illustration of LiPSs conversion mechanism on MXenes [71]. Copyright 2024, WILEY–VCH. **c** Li plating on bare Li and parallelly aligned MXene layers [73]. Copyright 2019, WILEY–VCH. **d** Band Structures of MXenes with various terminal groups [77]. Copyright 2024, American Chemical Society. **e**, **f** Binding energies, **g** PDOS, **h** ΔG of the conversion from Li_2_S_6_ to Li_2_S_2_, and **i**, **j** Li_2_S dissociation energy barrier on NSMX and MX surface[78]. Copyright 2024, Elsevier

Nazar’s group [67] was the first to use Ti_2_CT_x_-MXenes as sulfur hosts, demonstrating that LiPSs, initially adsorbed via S-Ti-C bonds, undergo conversion to Li_2_S. This process occurs through electron transfer via Ti_2_C or by disproportionation, leading to the formation of multiple Li_2_S nucleation sites on the surface (Fig. 3a). This pioneering work ignited widespread research into MXene-based materials for LSBs. Following this, various MXene materials have been developed, including Ti_3_C_2_T_x_/S conductive paper [68], flexible S@Ti_3_C_2_T_x_ electrodes [69], multilayered Ti_3_C_2_T_x_-polypropylene modified separators [70], and F-free Ti_3_C_2_ (Ff-Ti_3_C_2_) hosts [66]. These materials effectively capture soluble LiPSs via strong chemical interactions, converting them into thiosulfate and forming an in situ protective barrier to prevent the unwanted migration of LiPSs. Recently, Gogotsi and Oh et al. [71] systematically investigated the effects of different MXene types (Ti_2_CT_x_, Ti_3_C_2_T_x_, Ti_3_CNT_x_, Mo_2_TiC_2_T_x_, V_2_CT_x_, Nb_x_CT_x_, Nb_4_C_3_T_x_) on LiPSs adsorption using optical and spectroscopic methods. They discovered that all MXenes formed insoluble thiosulfate and polythionate complexes, with the adsorption and conversion of sulfur species varying by MXene type through disproportionation reactions (Fig. 3b). Notably, Ti_2_CT_x_ preferentially adsorbs Li^+^, while Mo_2_TiC_2_T_x_ effectively traps sulfur and converts LiPSs into Li_2_S_2_/Li_2_S.

MXenes, with their metallic conductivity and rapid Li^+^ diffusion, are increasingly recognized as promising materials for stabilizing Li anodes [72]. For example, lamellar Ti_3_C_2_T_x_ MXene layers were fabricated and adhered to the surface of a Li anode through a rolling technique, creating a smooth and dense protective layer (Fig. 3c) [73]. During cycling, Li tended to grow horizontally along the parallel-aligned MXene nanosheets, forming nucleation sites between the sheets, which facilitated uniform Li distribution. The inherent F-terminations in MXenes promoted the formation of a uniform and stable solid electrolyte interface (SEI) with Li-fluoride, effectively regulating the migration of Li^+^.

Under lean electrolyte conditions, the wettability of both sulfur cathode and Li anode is crucial for long-cycle performance. However, some negatively charged surface terminations (e.g., -F or -Cl) are unfavorable for wetting, leading to incomplete contact between MXenes and electrolyte [65]. Additionally, pristine MXenes face storage problems due to their instability in oxygen-rich environments [66]. To address these challenges, heteroatom doping, group grafting, and structural optimization have been explored to enhance the surface properties of MXenes [9, 74, 75]. These strategies aim to remove undesirable surface terminations or inhibit layer stacking, thereby improving structural stability and enhancing the adsorption capacity towards soluble LiPSs and Li^+^.

#### Heteroatom Doping

Heteroatom doping, including elements such as N, O, P, S, B, or I, has emerged as a highly effective strategy for enhancing the catalytic activity of MXenes [9], which can modify the original charge balance and electronic structure of the catalyst, resulting in charge rearrangement and a shift in the *d*-band center of metal sites. This alteration not only strengthens the interaction between sulfur species and the catalyst surface but also improves the overall catalytic performance by optimizing the adsorption and activation of sulfur species [76]. For instance, DFT calculations reveal that the Ti_3_C_2_I_2_ surface has a near-zero band gap (~ 0 eV), which is smaller than that of Ti_3_C_2_(OH)_2_ (0.006 eV), Ti_3_C_2_F_2_ (0.003 eV), Ti_3_C_2_O_2_ (0.127 eV), and Ti_3_C_2_Cl_2_ (0.034 eV) surfaces (Fig. 3d) [77]. This reduced band gap suggests that I-doping significantly alters the electronic structure. Furthermore, the binding energies between the I-doped Ti_3_C_2_T_x_ MXenes (I-MXene) and Li_2_S_8_, Li_2_S_6_, Li_2_S_4_, Li_2_S_2_, and Li_2_S species are -0.97, -0.87, -0.53, -1.49, -1.53, and -0.95 eV, respectively, indicating stronger chemisorption interactions with short-chain LiPSs. The I-MXenes effectively immobilized soluble LiPSs through strong Ti-S bonds and accelerated the reaction kinetics of LiPS conversion through enhanced charge transport. As a result, cells incorporating I-MXene-modified separators demonstrate good rate capability, delivering capacities of 1316, 886, 789, 723, and 655 mAh g^−1^ at 0.1C, 0.2C, 0.5C, 1C, and 2C, respectively. As reported, N/S co-doped MXenes (NSMX), synthesized via a thiourea-induced method, could further enhance adsorption effect for sulfur species by high binding energies (Fig. 3e, f) in LSBs [78]. Co-doping resulted in a shift of the Ti *d*-band center of NSMX from 1.54 eV in MX to 0.93 eV, bringing it closer to the Fermi level (Fig. 3g). This shift suggests that Ti sites in NSMX can transfer more electrons to LiPSs, thereby enhancing the electrocatalytic activity for the redox reactions of sulfur species. DFT calculations further showed that the NSMX surface exhibited smaller Gibbs free-energy changes (ΔG, 0.14 eV) during the liquid-to-solid conversion of Li_2_S_4_ to Li_2_S_2_ (Fig. 3h), as well as a lower Li_2_S dissociation barrier (0.41 eV), compared to the MX surface (Fig. 3i, j). Even under demanding conditions of high sulfur loading of 7.2 mg cm^−2^ and a low E/S ratio of 7.0 μL mg^−1^, the NSMX-based battery exhibited a remarkable reversible capacity of 729.9 mAh g^−1^ after 100 cycles, maintaining an exceptional average Coulombic efficiency of 99.7% at 0.2C. This superior electrochemical performance underscores the effectiveness of NSMX in promoting the complete conversion of massive sulfur species while mitigating the formation of low-activity “dead sulfur”.

A high-performance Li anode was developed by confining Li within S and N co-doped Nb_2_C MXene [79]. The doping with S and N enhanced both electroconductivity and lithiophilicity through the introduction of extrinsic defects and active sites. Compared to undoped Nb_2_C, the S and N co-doped Nb_2_C exhibited superior lithiophilicity due to their synergistic effects. This co-doped Nb_2_C could well serve as an effective 3D lithiophilic and conductive host, facilitating uniform nucleation and plating of Li metal. Additionally, the presence of heteroatoms expanded the interlayer spacing and stabilized the MXene structure, preventing pulverization and restacking during cycling. As a result, the Li metal anodes with co-doped Nb_2_C MXene showed excellent dendrite suppression, high coulombic efficiency (CE), extended lifespan, and outstanding performance in full cells.

#### Covalent Grafting

**Fig. 4:**
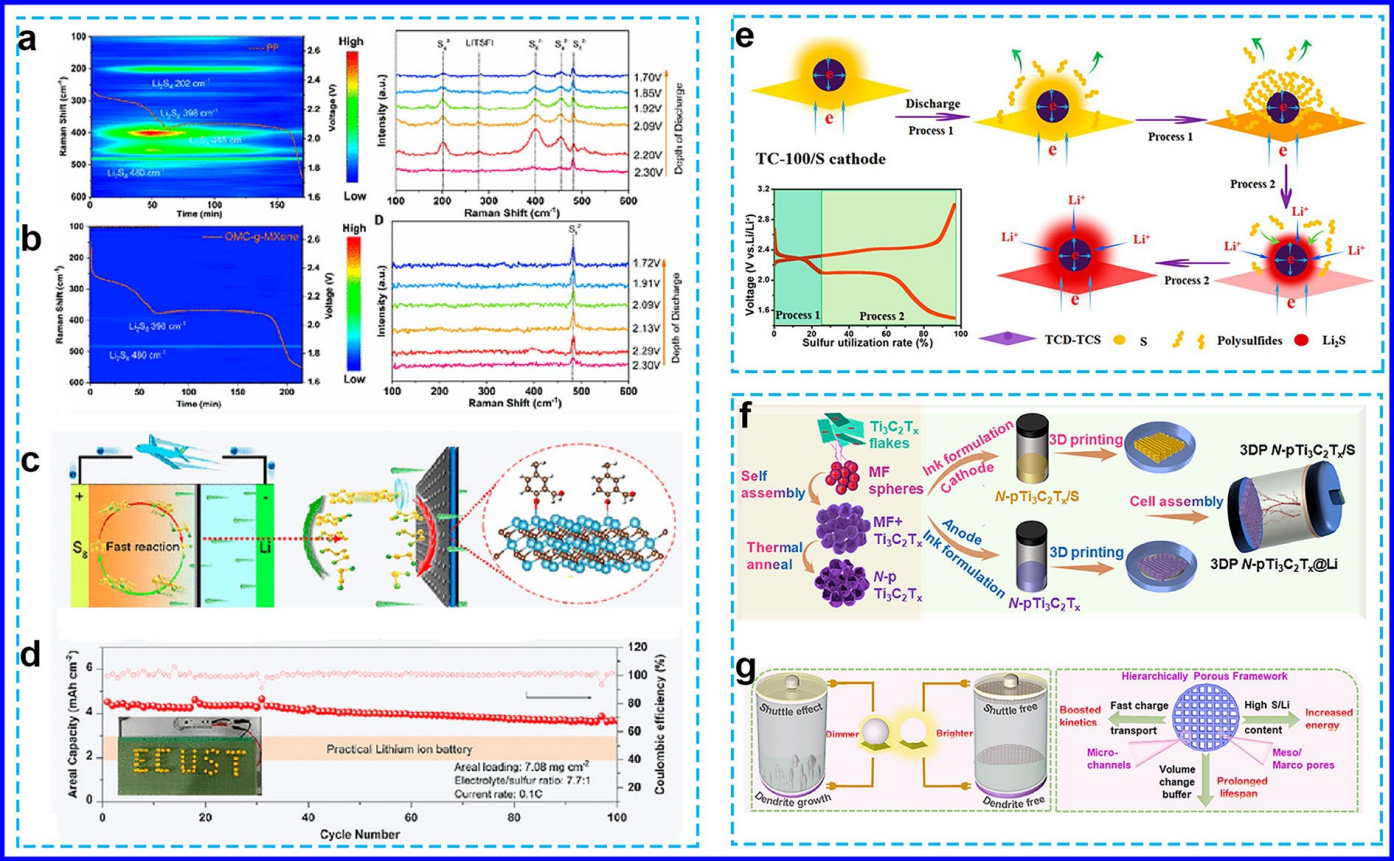
**a**, **b** In situ Raman images and spectroscopy of PP and OMC-g-MXene/PP separators, **c** illustration of the accelerated reaction kinetics of the OMC-g-MXene interlayer, and **d** cycle performances of the sulfur cathode based on OMC-g-MXene [83]. Copyright 2023, American Chemical Society. **e** Schematic illustration of redox reaction for TC-100/S cathodes [88]. Copyright 2019, American Chemical Society. **f** Schematic illustration of the preparation process and the employment of 3DP framework of N-pTi_3_C_2_T_x_, **g** dominant characteristic of porous 3DP framework in LSBs [89]. Copyright 2021, Elsevier

Covalent grafting effectively mitigates the restacking of MXene layers and introduces functional groups to immobilize LiPSs, such as guanidinium-based ionic-covalent organic nanosheets on Ti_3_C_2_ MXene nanosheets (GICOT) [80], microporous polymers grafted MXenes (MPGT) [81], and porous polydopamine layer coated MXenes (PPLT) [82]. If mesoporous carbons were uniformly grafted onto MXene nanosheets to form 2D heterostructure composites (OMC-g-MXene) [83], the resulting material would possess abundant defects and a carbon-coated layer. The in situ time-resolved Raman images (Fig. 4a and b) show that after introducing OMC-g-MXene into the system, no short-chain sulfur species are formed on the anodic side throughout the entire discharging process. Moreover, the OMC-g-MXene/PP separator exhibited the highest Li^+^ transport number of 0.89, significantly surpassing the control samples. This enhancement highlights the crucial role of OMC-g-MXene in facilitating Li⁺ kinetics in LSBs. The sulfur cathode based on OMC-g-MXene effectively promoted sulfur conversion and Li^+^ diffusion (Fig. 4c), which delivered a high areal capacity of 4.5 mAh cm^−2^ with a sulfur loading of 7.08 mg cm^−2^ and a 7.7 μL mg^−1^ E/S ratio (Fig. 4d).

#### Structural Optimization

2D MXenes can be engineered through structural optimization into 0D nanodots, 1D nanoribbons, and 3D nanoribbons networks. These structural designs enhance the exposure of active sites and optimize electron/ion transport pathways, offering more sulfur loading spaces, mitigating volume changes, and improving electrochemical reaction kinetics [84–87]. For instance, 0D Ti_3_C_2_T_x_ nanodots were successfully anchored onto 2D Ti_3_C_2_T_x_ nanosheets (TCD-TCS) via hydrothermal treatment with sodium alginate at 100 °C for 2 h, preventing layer restacking and nanodot aggregation [88]. The uniform distribution of ultrafine TCD on the TCS surface significantly reduced the interfacial resistance and allowed sulfur to tightly adhere to the TCD-TCS surface, enabling effective capture and conversion of high-concentration LiPSs (Fig. 4e). This structure also enhanced the structural integrity and tap density of the sulfur cathode during cycling. Additionally, Wu et al. [87] synthesized the interconnected α-Ti_3_C_2_ MNRs with highly conductive open macrospores, which facilitated efficient electrolyte diffusion and electron transport into the interior of electrode, significantly mitigating the shuttle effect of LiPSs. It was also reported that when the negatively charged Ti_3_C_2_T_x_ was wrapped around the positively charged polydopamine coated S spheres, a unique 3D free-standing Ti_3_C_2_T_x_ MXenes paper was formed. The lower plateau of the open-circuit voltage curve was 0.04 V higher than that of the closed-circuit voltage curve, indicating the rapid formation of solid-phase Li_2_S_2_ or Li_2_S. This phenomenon suggests that the 3D MXene framework effectively mitigates the uncontrolled diffusion of high concentration LiPSs through a dual immobilization mechanism, integrating thiosulfate/polythionate redox conversion with Lewis acid–base interactions.

Recently, extrusion-based 3D printing has emerged as a promising method for the scalable and customizable fabrication of energy storage devices. This technique enables the design of high-aspect-ratio structures within compact areas, which enhances the rapid diffusion of ions and electrons through thick electrodes [89]. For instance, an N-doped porous Ti_3_C_2_ MXene framework was developed via 3D printing. The resulting scaffold as both S and Li hosts offered hierarchical porosity, high conductivity, and abundant N-sites, showing excellent lithiophilic and sulfiphilic properties (Fig. 4f). The 3D-printed cathode, synthesized using a tailored sulfur ink, exhibited a highly porous and mechanically robust architecture, effectively accommodating high sulfur loading while facilitating efficient electron and ion transport. Concurrently, the incorporation of a 3D-printed MXene-based interlayer in the anode ensured uniform local current distribution and regulated Li deposition (Fig. 4g), achieving a low overpotential of 64 mV over 800 h at 5.0 mA cm^−2^/5.0 mAh cm^−2^. Moreover, the fully 3DP-LSB demonstrated remarkable cycling stability, delivering an high areal capacity of 8.47 mAh cm^−2^ under a high sulfur loading of 12.02 mg cm^−2^ after 60 cycles.

Table 1 summarizes detailed battery performance data and compares the relative properties of carbon-based materials, emphasizing that heteroatom doping, group grafting, and the structural optimization effectively enhance the electrochemical properties of pristine MXenes in high-sulfur loading and lean electrolyte LSBs. However, precise control over the quantity and type of dopants or grafted materials remains a significant challenge. Moreover, excessive thickness or mass of these interlayers can hinder Li^+^ diffusion and increase the overall weight of the battery, thereby impairing its energy efficiency.

**Table 1:** Cycling performances of LSBs based on carbon-based materials and MXenes as active materials

Samples	Sulfur loading (mg cm^−2^)	E/S ratio (μL mg^−1^)	Current density /cycling	Specific capacity (mAh g^−1^)	Area capacity (mAh cm^−2^)	Attenuation rate (%)	Rate performance (mAh g^−1^)	Refs
Ti_2_CT_x_-MXenes	1.0	50.0	0.5C/100	960.0	0.85	92	1200 (0.2C), 1090 (0.5C), 1000 (1.0C)	[67]
Ti_3_C_2_T_x_	2.5	–	1.0C/1500	970.0	2.42	82.98	1383 (0.1C), 1270 (0.5C), 1196 (1.0C), 1075 (2.0C)	[68]
Flexible Ti_3_C_2_T_x_	~ 2.5	–	2.0C/175	1170.0	2.92	93.9	1350 (0.1C), 1335 (0.2C), 1280 (0.5C), 1238 (1.0C), 1170 (2.0C)	[69]
Ti_3_C_2_T_x_-polypropylene	1.2	20.0	1.0C/200	630.0	0.76		260 (0.05C), 235 (0.1C), 185 (0.2C), 150 (0.5C), 127 (1.0C), 119 (2.0C)	[57]
Ff-Ti_3_C_2_	5.8	3.5	0.5C/35	737.0	4.27	75.8	1312 (0.1C), 1133 (0.2C), 972 (0.5C), 849 (1.0C), 771 (2.0C), 690 (3.0C)	[66]
I-MXene	5.0	~ 15.0	0.2C/200	578.0	2.89	75.9	1128 (0.1C), 761 (0.2C), 652 (0.5C), 601 (1.0C), 555 (2.0C)	[77]
NSMX	7.2	7.0	0.2C/100	947.2	5.25	76	1315 (0.2C), 1041 (0.5C), 922 (1.0C), 795 (2.0C), 712 (3.0C), 649 (4.0C), 595(5.0C)	[78]
GICOT	7.6	–	0.1C/200	1092.0	8.29	85.3	1417 (0.05C), 1280 (0.1C), 1186 (0.2C), 1048 (0.5C), 956 (1.0C), 846 (2.0C), 771 (3.0C), 687 (5.0C)	[80]
MPGT	1.0	30.0	0.5C/1000	508.0	0.51	55.46	1134 (0.2C), 916 (0.5C), 853 (1.0C), 730 (2.0C), 700 (3.0C), 610 (4.0C)	[81]
PPLT	5.0	-	0.2C/1000	651.0	3.26	71.4	1126 (0.1C), 912 (0.2C), 886 (0.5C), 795 (1.0C), 600 (2.0C), 442 (4.0C)	[82]
OMC-g-MXene	7.1	7.7	0.1C/100	635.6	4.50	55.7	1142 (0.1C), 986 (0.2C), 952 (0.5C), 795 (1.0C), 744 (2.0C), 537 (3.0C)	[83]
TCD-TCS	9.2	–	0.05C/100	826.0	7.60	62.7	1389 (0.05C), 1377 (0.2C), 1183 (0.5C), 1081 (1.0C), 950 (2.0C), 882 (3.0C)	[88]
α-Ti_3_C_2_ MNRs	4.0	–	0.3C/200	1229.0	4.91	94	1560 (0.12C), 1272 (0.3C), 1127 (0.6C), 1042 (0.9C), 992 (1.2C), 944 (1.8C), 895 (2.4C), 854 (3.0C)	[87]
3D-printed cathode	12.02	5.0	0.2C/150	914	8.47	72.6	1283 (0.2C), 1147 (0.5C), 1001 (1.0C), 827 (2.0C)	[89]
CNT	3.5	-	1.17 mA cm^−2^/100	670	3.6	64.9	-	[90]
3DG	4.7		0.5C/170	979	6.4	89	1051 (0.05C), 921 (0.1C), 893 (0.2C), 763 (0.5C), 721 (1.0C), 450 (2.0C)	[91]
Ti_3_C_2_T_*x*_/rGO	2	10	1.5C/500	740	1.5	78.4	1480 (0.1C), 1280 (0.2C), 1100 (0.5C), 880 (1.0C),660 (2.0C)	[92]
CNT/NG	6.3	–	0.05C/160	700	2.3	70.4	1114 (0.05C), 824(0.1C), 792 (0.2C)	[93]
CNT-Ti_2_C	5	7	0.5C/1200	450	5	47.4	-	[94]
N-Ti_3_C_2_T_x_	5.1	6	0.2C/500	588	1.69	77	1083(0.2C), 947 (0.5C), 835 (1.0C), 770 (2.0C),	[95]
CGS	1.1	–	0.5C/500	737.8	1.67	89	1346 (0.1C), 1155 (0.2C), 1024 (0.5C), 931 (1.0C), 827 (2.0C), 606 (5.0C), 535 (10.0C)	[96]

### MXenes as Substrates

MXenes can serve as substrates to support other materials. The integration of MXenes with other materials offers intrinsic advantages that are difficult to achieve with single-component materials, especially in LSBs [11, 27, 97–99]. The *d*-band theory, widely utilized to explain the catalytic behavior of materials, provides essential insights into the mechanisms governing catalysis [100]. An upshift in the metal *d*-band center elevates the antibonding orbitals involved in the *d*–*p* hybridization between metal atoms in the catalyst and sulfur atoms in LiPSs. This shift strengthens the adsorption of LiPSs, thereby enhancing the catalytic performance [101, 102]. To optimize the catalytic properties of MXenes-based electrocatalysts, strategies such as manipulating lattice strain, doping, defect engineering, and seeding single-atom catalysts are commonly employed to optimize the *d*-band center. Furthermore, methods like constructing built-in electric fields and inducing "cocktail effect" have further enhanced the electrocatalytic activity of MXenes-based electrocatalysts.

#### Manipulating Lattice Strain

**Fig. 5:**
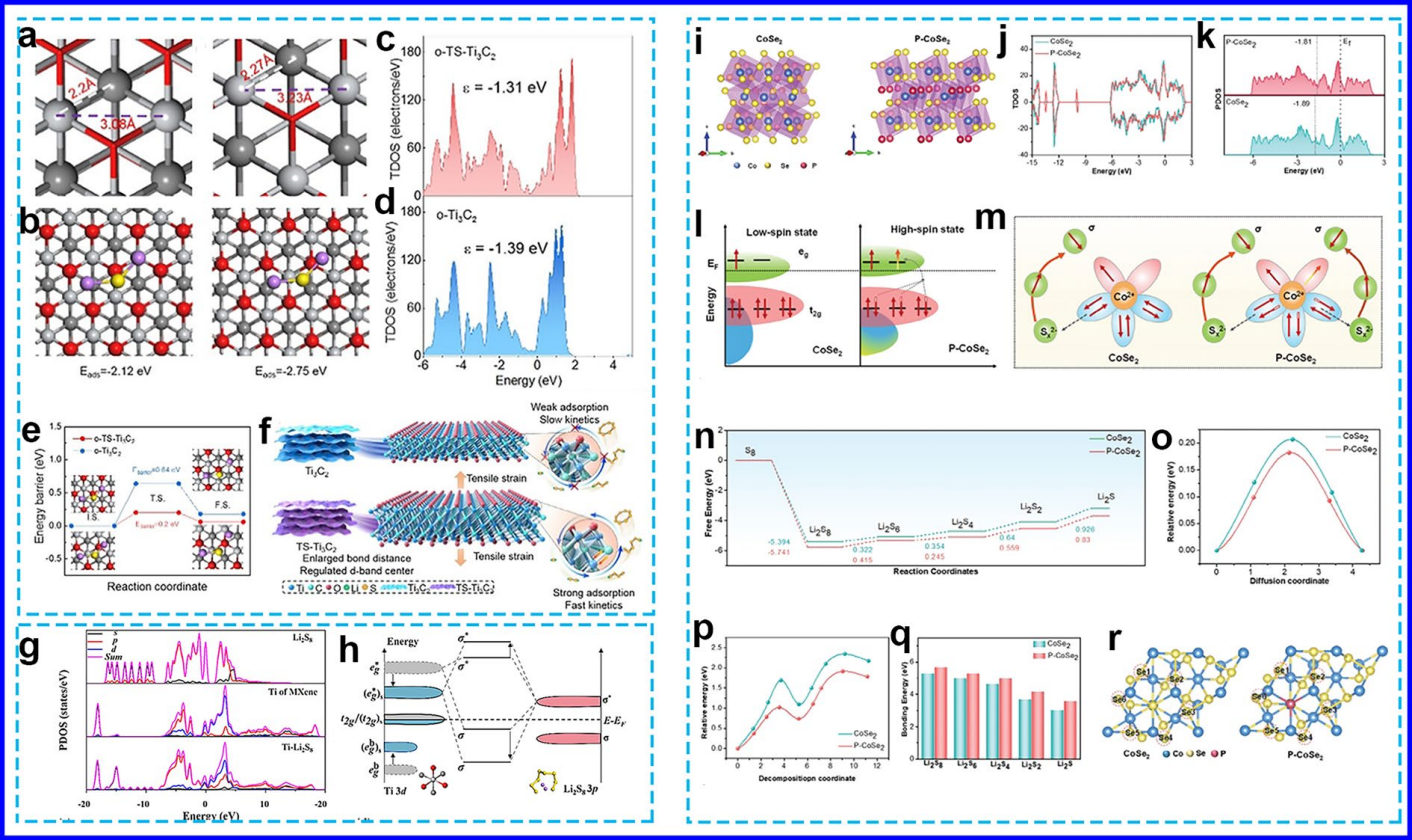
**a**, **b** Crystal structure and bond length, **c**, **d** PDOS of Ti-3*d* orbitals, **e** Li_2_S dissociation energy barrier, and **f** reaction mechanism on Ti_3_C_2_ and TS-Ti_3_C_2_ surfaces in LSBs [108]. Copyright 2021, WILEY–VCH. **g** PDOS and **h** orbital interactions between Ti-3*d* orbitals of MXene and S-3*p* orbitals of Li_2_S_8_ [109]. Copyright 2022, Elsevier. **i** Crystal structures, **j** TDOS plots, **k** PDOS, **l** energy band, **m** electronic coupling, **n** ΔG profiles from S_8_ to Li_2_S, **o** Li^+^ diffusion energy profile, **p** Li_2_S decomposition energy profiles, **q** binding energies of various sulfur species, and **r** the Bader charge for CoSe_2_ and P-CoSe_2_ [110]. Copyright 2024, WILEY–VCH

Strain refers to the deformation that occurs when a crystal is subjected to compression, tension, or shear forces. By manipulating the surface strain of catalysts, whether tensile or compressive, changes in atomic bond lengths or lattice mismatches can be induced, resulting in alterations to their electronic structure and catalytic properties [103, 104]. Tensile or compressive lattice strain can shift the *d*-band center, which plays a crucial role in determining the adsorption and desorption behaviors of intermediates on catalysts [105–107]. For instance, Chen and Wang et al. [108] developed a 3D microporous electrocatalyst as a multifunctional sulfur immobilizer and promoter, consisting of tensile-strained MXene nanosheets interwoven with carbon nanotube (CNT) tentacles (MXene/CNT). During the spray-drying process, a surface oxidation layer was formed in situ on the MXenes, resulting in anion substitution and the formation of an O-Ti-C interface. This oxidation induced internal stress on the surface, leading to lattice distortion and the enlargement of Ti–Ti bonds. The mismatch between the Ti_3_C_2_ layer and the oxidation layer (O-Ti_3_C_2_) created tensile strain at the interface. DFT calculations revealed that the O-Ti_3_C_2_ induced a 5% lattice tensile strain (O-TS-Ti_3_C_2_), which significantly expanded the bond lengths (Fig. 5a, b). These increased atomic spacing weakened atomic interactions and resulted in a narrower band gap and a shifted *d*-band center (− 1.31 eV), closer to the Fermi level than O-Ti_3_C_2_ (Fig. 5c, d). This tensile strain effect promoted the surface adsorption and catalytic conversion of high concentration LiPSs and Li_2_S oxidation via reducing the Li_2_S cluster decomposition energy barrier from 0.64 to 0.2 eV (Fig. 5e), accelerating their transformation process (Fig. 5f). Moreover, the MXene/CNT interconnected framework not only exposed vast electrode/electrolyte interface but also established an open but robust structure, offering potent sulfur immobilization, strong LiPS confinement and admirable structure stability.

Zhao's group [109] also reported a strain-regulation strategy for enhancing the catalytic performance of MXenes in LiPSs conversion. The thickness of MXene decreases as compressive strain increases (from 0 to 6%), which deforms the TiC_3_O_3_ octahedral configuration. However, under 7% strain, the MXene structure collapses due to thermodynamic instability. At 6% compressive strain, the adsorption energy of soluble LiPSs was − 1.5 eV, about three times higher than that of MXenes without strain. Projected density of states (PDOS) analysis shows that the S-3*p* orbital of Li_2_S_8_ and the Ti-3*d* orbital of MXenes overlap after adsorption, resulting in a decrease in the S-3*p* orbital energy and an increase in the Ti-3*d* orbital energy (Fig. 5g, h), indicating that Li_2_S_8_ was chemically bound to MXenes. The 6% strained MXenes optimally accelerated the conversion rate and suppressed the shuttle effect of LiPSs. Based on these theoretical insights, authors synthesized strain-induced wrinkle flower-shaped MXene (w-MXene), which not only exhibited strong adsorption for high-concentration LiPSs but also accelerated their transformation. This resulted in an impressive initial areal capacity of 16.53 mAh cm^−2^ under high sulfur loading and demonstrated long-cycle stability.

Recently, Wang et al. [110] found that incorporating P atoms into the mixed-phase cubic and orthorhombic CoSe_2_ (P-CoSe_2_) on grown 3D crumpled MXene (P-CoSe_2_/MXene) induced a phase transformation to a pure orthorhombic structure, which generated tensile strain and enhanced charge localization due to the elongated Co-Se bond length. The total DOS of P-CoSe_2_ revealed a stronger density of states near the Fermi level compared to pure CoSe_2_ (Fig. 5i, j), indicating increased electronic conductivity. The PDOS further showed that the *d*-band center of Co shifted from − 1.89 to − 1.81 eV (Fig. 5k), likely due to the tensile stress caused by the elongated Co-Se/P bond distance. The partial replacement of Se with P increases the Se-Se/P bond distance, reducing the bonding-antibonding splitting. This allowed the t_2g_ electron to easily transfer into the e_g_ orbital, modulating the spin states of the Co center (Fig. 5l, m). The P-CoSe_2_/MXene structure showed more exposed *d*-electron pairs and unpaired electrons than cubic CoSe_2_. The upshift of the *d*-band center, coupled with the enhanced Bader charge at Se sites, synergistically facilitated dual coordination with both Li and S sites in LiPSs with low Gibbs free energy (0.83 eV) of RDS and Li_2_S decomposition activation energy (1.9 eV) and small Li^+^ diffusion barrier (0.18 eV) (Fig. 5n–r). Under conditions with a sulfur areal loading of 4.0 mg cm^−2^ and an E/S ratio of 10 μL mg^−1^, the P-CoSe_2_/MXene cells achieved a high initial areal capacity of 3.6 mAh cm^−2^.

#### Doping or Defect Engineering

**Fig. 6:**
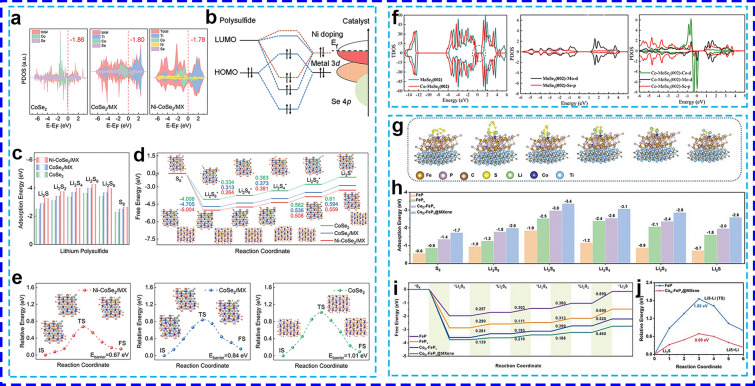
**a** PDOS, **b** orbital interactions, **c** binding energies, **d** ΔG curves, and **e** Li_2_S decomposition energy barriers on various surfaces [114] Copyright 2024, WILEY–VCH. **f** TDOS and PDOS of various surfaces [111]. Copyright 2021, American Chemical Society. **g** The optimized adsorption structures, **h** calculated binding energies,** i** ΔG profiles from S_8_ to Li_2_S, and **j** Li_2_S decomposition barriers profiles [115]. Copyright 2023, WILEY–VCH

Doping metal heteroatoms into metal-based catalysts enhances both the electronic structure and metallic conductivity. Sometimes, doping modification may introduce abundant defects that expose additional reactive sites for catalytic reactions [111]. These defects effectively modulate the electrocatalytic properties at the atomic level [112, 113]. This doping or defect engineering accelerates redox kinetics and suppresses the shuttle effect of LiPSs, thereby improving the overall catalytic performance. For example, Ni-doped CoSe_2_ nanoparticles were uniformly integrated onto the surface of hollow MXene to form the Ni-CoSe_2_/MX [114]. In this structure, a small proportion of Co atoms within the CoSe_2_ crystal lattice were substituted by Ni^2+^ ions. This doping induced a significant upshift in the *d*-orbital center relative to the Fermi level in Ni-CoSe_2_/MX, which exhibited an upshifted *d*-band center of -1.78 eV after Ni doping, compared to -1.80 eV in CoSe_2_/MX (Fig. 6a). This upshifts resulted in a higher filling fraction of the lowest unoccupied molecular orbital (LUMO) of LiPSs (Fig. 6b), thereby promoting the hybridization between the transition metal sites and LiPSs. As expected, Ni-CoSe_2_/MX delivered higher bind energies (Fig. 6c), a lower ΔG of 0.559 eV compared to CoSe_2_/MX (0.594 eV) and CoSe_2_ (0.61 eV) at the transformation from Li_2_S_2_ to Li_2_S (Fig. 6d), and reduced Li_2_S energy barrier (from 0.84 to 0.67 eV) (Fig. 6e). Moreover, the prevention of MXene self-restacking ensured maximum exposure of Ni-CoSe_2_ nanoparticles, which provided additional active sites. This enhancement facilitated stronger adsorption of sulfur species and improved catalytic effects for the conversion of high-concentration LiPSs and the decomposition of Li_2_S.

Similarly, Li et al. [111] designed a bifunctional electrocatalyst by doping Co into MoSe_2_, creating Co-MoSe_2_, which was then in situ hybridized with conductive MXene nanosheets through a one-step hydrothermal reaction to form Co-MoSe_2_/MXenes. The introduction of Co into the basal plane and edge of MoSe_2_ led to a noticeable shift of both the conduction and valence bands towards the Fermi level, narrowing the band gap from 1.1 to 0.5 eV (Fig. 6f). This modification also resulted in substantial movement of the Co-*d*, Mo-*d*, and Se-*p* orbitals towards the Fermi level, particularly the Se-*p* orbitals, whose intensity increased fivefold due to the strong interaction with the Co atom. As a result, Co doping induced structural disorder and defects, leading to an increase in the number of catalytic active sites for the adsorption and conversion of LiPSs as well as the oxidation of Li_2_S, which in turn accelerated the redox kinetics. The dense S/Co-MoSe_2_/MXene monolith cathode demonstrated outstanding rate performance, delivering capacities of 1454, 1390, 1290, 1170, 995, and 759 mAh g^−1^ at 0.1C, 0.2C, 0.5C, 1C, 2C, and 5C, respectively.

Additionally, Tang and Sun et al. [115] reported the development of a new family of dual-defect catalysts, CoD-FeP_v_@MXene, which incorporates P vacancies and Co doping in FeP on MXene. The introduction of both Co doping and P vacancies lowered the binding energy of LiPSs on the catalyst surface, facilitating stronger adsorption and capture of LiPSs (Fig. 6g, h). Furthermore, the CoD-FeP_v_@MXene exhibited the lowest free energy barrier (0.46 eV) for the reduction of Li_2_S_2_ to Li_2_S (Fig. 6i) and the lowest decomposition energy barrier (0.69 eV) for Li_2_S (Fig. 6j), compared to other catalysts. This promoted the nucleation and decomposition of Li_2_S. The P vacancies provided additional active sites for LiPS adsorption, while Co doping generated a local electric field that lowered the reaction energy barrier and accelerated Li_2_S dissolution. The synergistic effects of these vacancies and heteroatom doping not only suppressed the shuttle effect but also improved the utilization of sulfur, leading to enhanced rate performance and cycling stability.

#### Seeding Single-Atom Catalysts

**Fig. 7:**
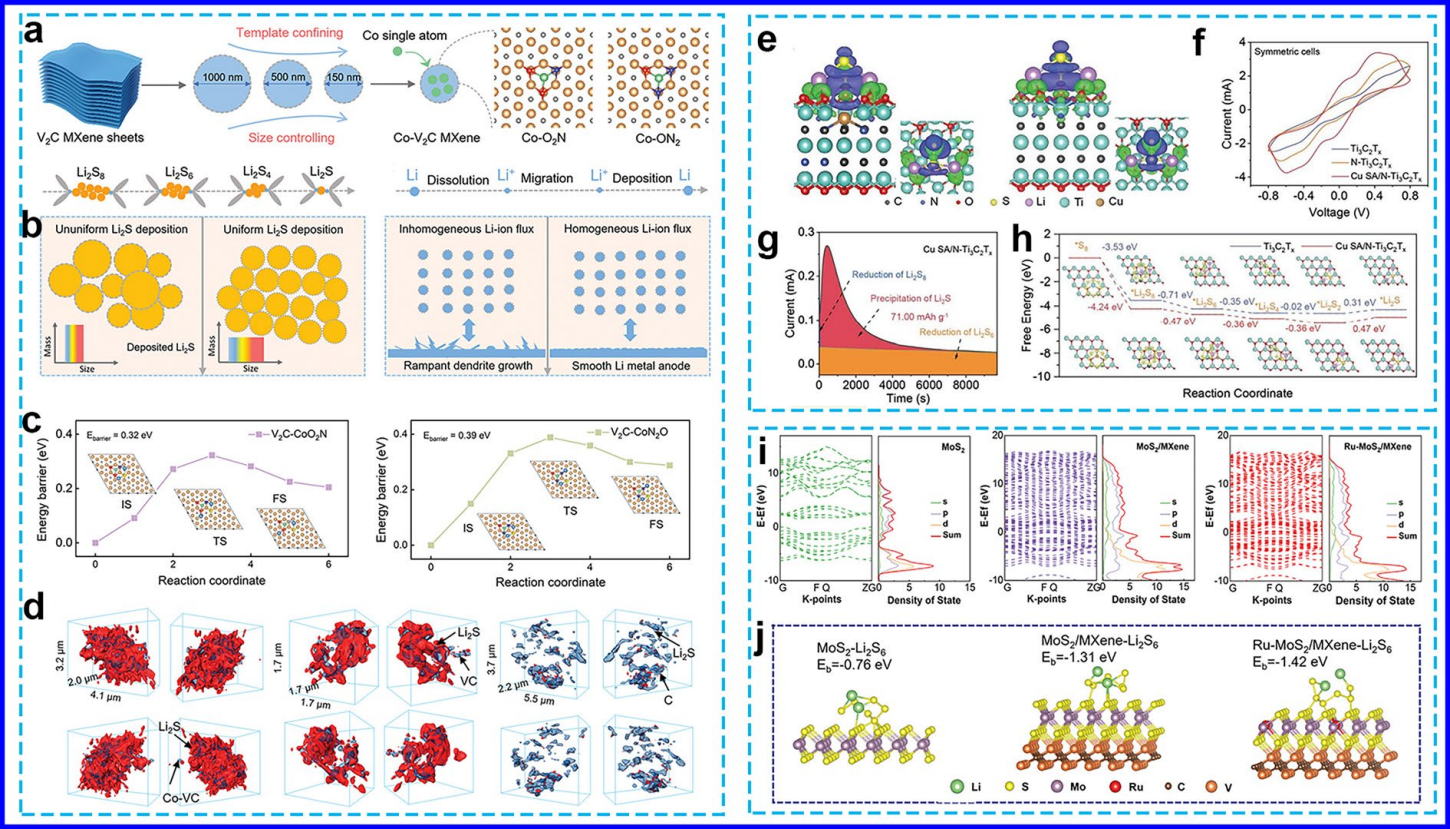
**a** Schematic illustration of the preparation process, **b** optimizing S cathode and Li anode for Co-VC, **c** Li_2_S dissociation energy barriers, and **d** synchrotron radiation X-ray 3D nano-CT images of Li_2_S deposition on various substrates [116]. Copyright 2024, WILEY–VCH. **e** Charge density, **f** CV curves, **g** Li_2_S deposition curves,** h** ΔG profiles from S_8_ to Li_2_S [117]. Copyright 2023, WILEY–VCH. **i** Calculated band structure and PDOS, and **j** binding energies between Li_2_S_6_ and various surfaces [99]. Copyright 2024, WILEY–VCH

Single-atom catalysts (SACs) have attracted considerable attention due to their superior catalytic activity and well-defined active sites. These attributes offer significant advantages, particularly in accelerating sulfur redox kinetics in Li–S catalysis. The synergistic integration of SACs with MXenes represents a promising avenue for the development of novel physicochemical properties. Cai et al. [116] demonstrated the decoration of atomically dispersed Co sites on V_2_C MXene with a size-effect optimization (Co-VC), where isolated Co atoms form stable binary coordination with O and N atoms, such as Co-O_2_N and Co-ON_2_. The high atom utilization efficiency (~ 100%) and the diverse coordination environment of Co atoms, combined with the size-effect-optimized VC substrate, significantly enhance the catalytic activity for both S and Li conversion reactions. In the S cathode, this rational design effectively guides the nucleation and growth of Li_2_S, resulting in a Li_2_S product with higher mass, smaller size, and improved homogeneity (Fig. 7a). For the Li anode, the Li plating/stripping behaviors are optimized by controlling the Li^+^ flux, ensuring an ideal working surface (Fig. 7b). This optimization is achieved by modulating the adsorption and diffusion of Li^+^ on Co-VC, benefiting from ultrafast Co atom utilization. Consequently, the Co-VC heterostructure promoted more efficient Li_2_S evolution with a lower energy barrier value of 0.32 eV (Fig. 7c, d).

Similarly, a series of metal single atoms (denoted as M SA/N-Ti_3_C_2_T_x_, where M represents Cu, Co, Ni, Mn, Zn, In, Sn, Pb, and Bi) were immobilized on N-doped Ti_3_C_2_T_x_ using a vacancy-assisted approach [117]. DFT calculation identified that the Cu–N_1_C_2_ coordination as the active sites exhibited higher binding energy and larger electron clouds than pristine Ti_3_C_2_T_x_, enhancing the interaction for LiPSs by single Cu sites (Fig. 7e). CV and Li_2_S deposition curves proved that Cu SA/N-Ti_3_C_2_T_x_ could promote LiPSs conversion and Li_2_S deposition (Fig. 7f, g). The discharge process was more thermodynamically favorable on Cu SA/N-Ti_3_C_2_T_x_ than on Ti_3_C_2_T_x_. The energy barrier for the conversion of Li_2_S_4_ to Li_2_S on Cu SA/N-Ti_3_C_2_T_x_ was 0.11 eV, lower than the 0.29 eV barrier on Ti_3_C_2_T_x_ (Fig. 7h), indicating that Cu SA/N-Ti_3_C_2_T_x_ facilitates the kinetic conversion of LiPSs. Moreover, a triple-boundary heterostructure composed of MXene decorated with Ru-doped, defect-rich 1 T/2H MoS_2_ was also synthesized [99]. The DOS of the Ru–MoS_2_/MXene composite was higher than that of MoS_2_ or MoS_2_/MXene alone (Fig. 7i), indicating increased electron density and enhanced electrical conductivity. The calculated binding energy of Ru-MoS_2_/MXene was − 1.42 eV, stronger than MoS_2_ (− 0.76 eV) and MoS_2_/MXene (− 1.31 eV) (Fig. 7j), suggesting that Ru-MoS_2_/MXene has superior LiPS adsorption capacity, effectively mitigating the shuttle effect. Moreover, MoS_2_/MXene could prevent the desorption of solid Li_2_S_2_/Li_2_S from the host, thereby passivating the catalyst and inhibiting further conversion.

#### Constructing Built-in Electric Field

MXenes exhibit metallic conductivity, making them highly amenable to forming heterostructures when combined with other materials. When two materials with distinct Fermi levels come into contact, a discontinuity in the Fermi levels occurs at the interface [48]. This discrepancy induces the formation of a polarization interphase, accompanied by a potential energy difference [59]. The resulting energy gradient drives the spontaneous migration of free electrons until the Fermi levels of the two materials equilibrate [118]. As a result, electrons accumulate in one region, creating an electron-rich zone, while electron holes remain in the opposite region, ultimately generating a built-in electric field (BIEF) at the interphase [59]. In LSBs, the BIEF in catalysts can affect the electronic structure, surface adsorption, and catalytic activity toward LiPSs.

**Fig. 8:**
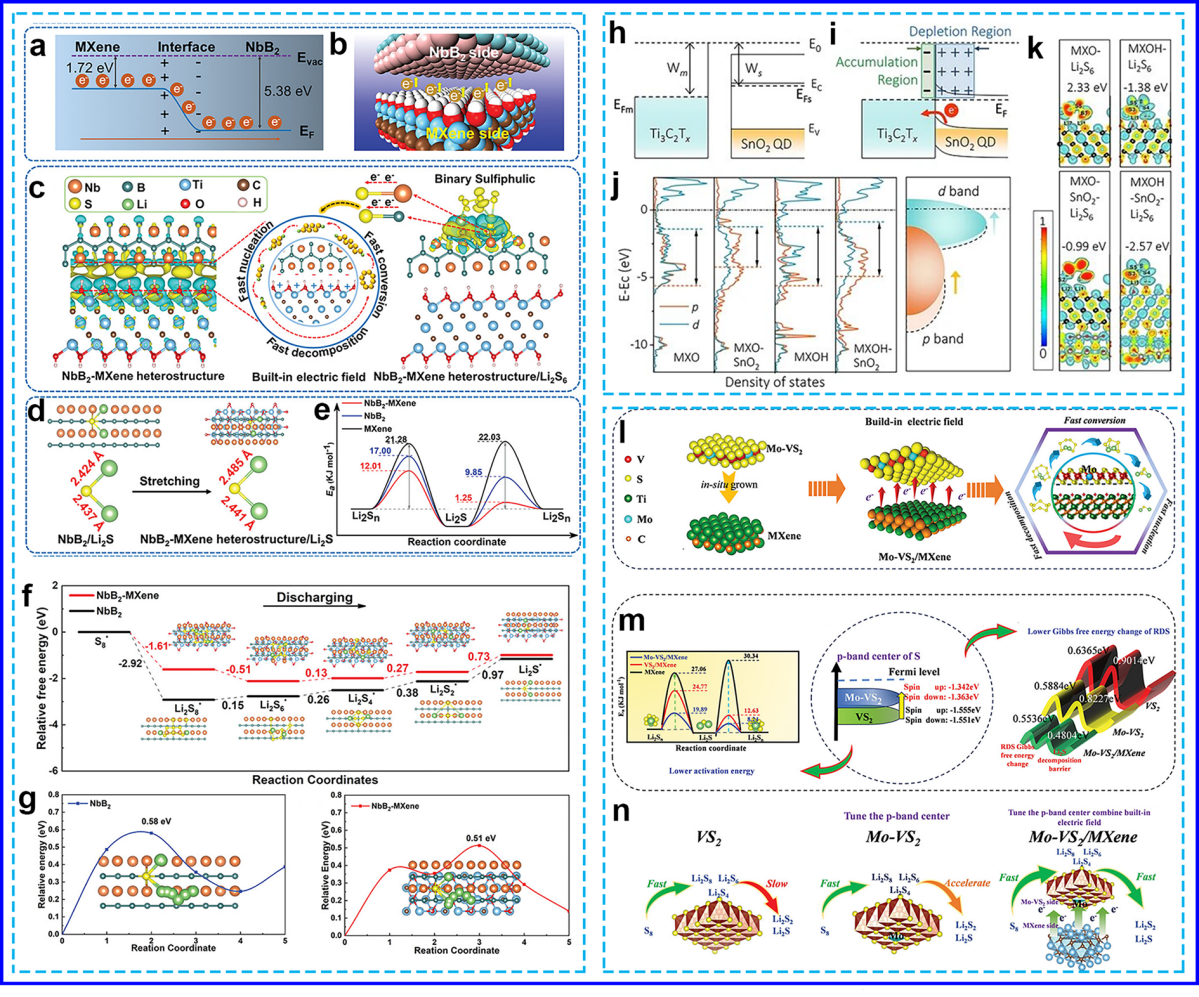
**a**, **b** Electron redistribution, **c** charge density difference, **d** Li_2_S adsorbed and the bond length of Li–S bond, **e** the activation energy for LiPSs/Li_2_S conversion, **f** ΔG profiles from S_8_ to Li_2_S, and **g** Li_2_S decomposition path on various surfaces [59]. Copyright 2023, WILEY–VCH. **h** Energy band diagram between SnO_2_ and MXene, **i** electron localization functions, **j** PDOS analysis, and **k** binding energies [119]. Copyright 2024, Springer Nature. **l** The formation of BIEF, **m** activation energy, p-band center, and ΔG profiles, and **n** LiPSs conversion process on different surfaces [124]. Copyright 2024, WILEY–VCH

Highly conductive binary sulfiphilic NbB_2_-MXene heterostructures were strategically designed to generate a BIEF through a simple one-step borothermal reduction process [59]. The Fermi level (*E*_F_) of NbB_2_ (− 5.38 eV), measured relative to the vacuum level (*E*_vac_), is significantly lower than that of MXene (− 1.72 eV) (Fig. 8a). When these materials are brought into contact, the potential energy difference drives the spontaneous migration of free electrons from MXene to the NbB_2_ side until the Fermi levels of both materials equilibrate (Fig. 8b). As a result, electrons accumulate at the NbB_2_ site, while electron holes accumulate at the MXene site, leading to the formation of a BIEF between NbB_2_ and MXene (Fig. 8c). The charge density difference further confirms the charge redistribution at the interface, where electrons are concentrated at NbB_2_ sites, and holes are concentrated at the MXene side, reinforcing the presence of the BIEF. This electron redistribution endows the NbB_2_-MXene heterostructure with moderate adsorption properties for LiPSs (Fig. 8d), as Nb and B atoms, having gained more electrons, weaken their strong adsorption to LiPSs. The D_Li_^+^ of the cell with S/NbB_2_-MXene is 6.3 × 10^−8^ cm^2^ s^−1^, which is significantly higher than that of S/NbB_2_ (3.3 × 10^−8^ cm^2^ s^−1^) and S/MXene (2.3 × 10^−8^ cm^2^ s^−1^), demonstrating that the BIEF effectively accelerates the Li^+^ diffusion rate. The charge redistribution and boundary defects within the heterostructure expose additional active sites, thereby reducing the free energy barrier for Li_2_S_2_ to Li_2_S (0.73 eV) (Fig. 8e) and for Li_2_S decomposition (0.51 eV) (Fig. 8f). This facilitates the enrichment of chemical anchor sites and catalytic centers, which, in turn, enhances the redox kinetics of LiPS conversion, even under high-sulfur loading in the cathode.

Zhang et al. [119] also reported the creation of an ultrathin SnO_2_@MXene heterostructure, where SnO_2_ quantum dots (QDs) are uniformly distributed across a MXene layer. When metallic Ti_3_C_2_T_x_ MXene contacts semiconducting SnO_2_, electron transfer from SnO_2_ to MXene occurs to balance their Fermi levels (Fig. 8g). This electron transfer leads to the formation of a depletion region on the SnO_2_ side (Fig. 8h), which carries a positive charge, and an accumulation region on the MXene side, which carries a negative charge. As a result, a BIEF is generated, promoting electron flow across the interface. This charge redistribution alters the coordination environment of the electron-rich and electron-deficient regions, affecting the electronic structure, surface adsorption with high binding energy of − 2.57 eV, and catalytic activity toward LiPSs (Fig. 8i, j). The introduction of highly catalytic heterojunction sites significantly lowers the nucleation energy, promoting more efficient nucleation without a noticeable barrier, even under conditions of high sulfur loading. Various similar heterostructures, such as TiO_2_-MXenes [120], MnO_x_/MXenes [121], SnS_2_-MXene [11], MXene/1 T-2H MoS_2_-C [122], and MoS_2_@Mo_2_C MXene [41], have been developed to enhance the performance of LSBs.

In another study, when VS_4_ contacts SnS_2_, the work function difference between SnS_2_ and VS_4_ causes a depletion of charge near the V atom in VS_4_, while charge accumulates at the S atom in SnS_2_ [123]. This electron transfer forms a conductive path from VS_4_ to SnS_2_, which is further facilitated by the metallic-like behavior of MXenes. The outer electrons of Ti in MXenes are prone to being lost, and when MXenes contacts the n-type semiconductor VS_4_, an ohmic contact is established. This allows for the spontaneous coupling of Ti’s outer electrons to the sulfur atoms in VS_4_, creating a rapid electrical pathway nearly independent of resistance. These findings suggest that the double heterostructure formed by MXene-VS_4_-SnS_2_ accelerates electron movement and participates in the catalytic transformation of LiPSs, reducing the activation energy for LiPSs conversion and lowering the decomposition barrier of Li_2_S. Electrochemical tests show that the MXene-VS_4_-SnS_2_ structure exhibits enhanced cycling stability and high performance. Besides, Li et al. [124] also proposed a strategy that combines cation-doping engineering and the BIEF effect to modulate the *p*-band centers of active sites and enhance interfacial charge transport in heterojunctions (Fig. 8k). Specifically, Mo-doped VS_2_ nanosheets were grown in situ on a MXene surface to form a Mo-VS_2_/MXene heterojunction. The Mo-doping shifts the p-orbital energy of sulfur atoms in VS_2_ toward the Fermi level, strengthening the S-Li bonding and improving the adsorption of LiPSs (Fig. 8l). This increases the efficiency of SRR by reducing the activation energy and the energy barriers associated with the conversion of LiPSs. The BIEF effect at the hetero-interface facilitates spontaneous electron rearrangement, which accelerates electron transfer and enhances the thermodynamic and kinetic properties of SRR (Fig. 8m), thereby alleviating the shuttle effect of LiPSs. The Mo-VS_2_/MXene heterojunction exhibited significantly improved SRR catalytic performance compared to pure VS_2_ or VS_2_/MXene (Fig. 8n). Furthermore, the Li_2_S decomposition barrier on the Mo-VS₂/MXene surface (0.48 eV) is significantly lower than those on VS_2_ (0.90 eV) and Mo-VS_2_ (0.82 eV), highlighting the superior capability of the Mo-VS₂/MXene catalyst in facilitating Li_2_S transformation. This suggests its potential as an efficient bifunctional electrocatalyst for LSBs.

#### Inducing Cocktail Effect

**Fig. 9:**
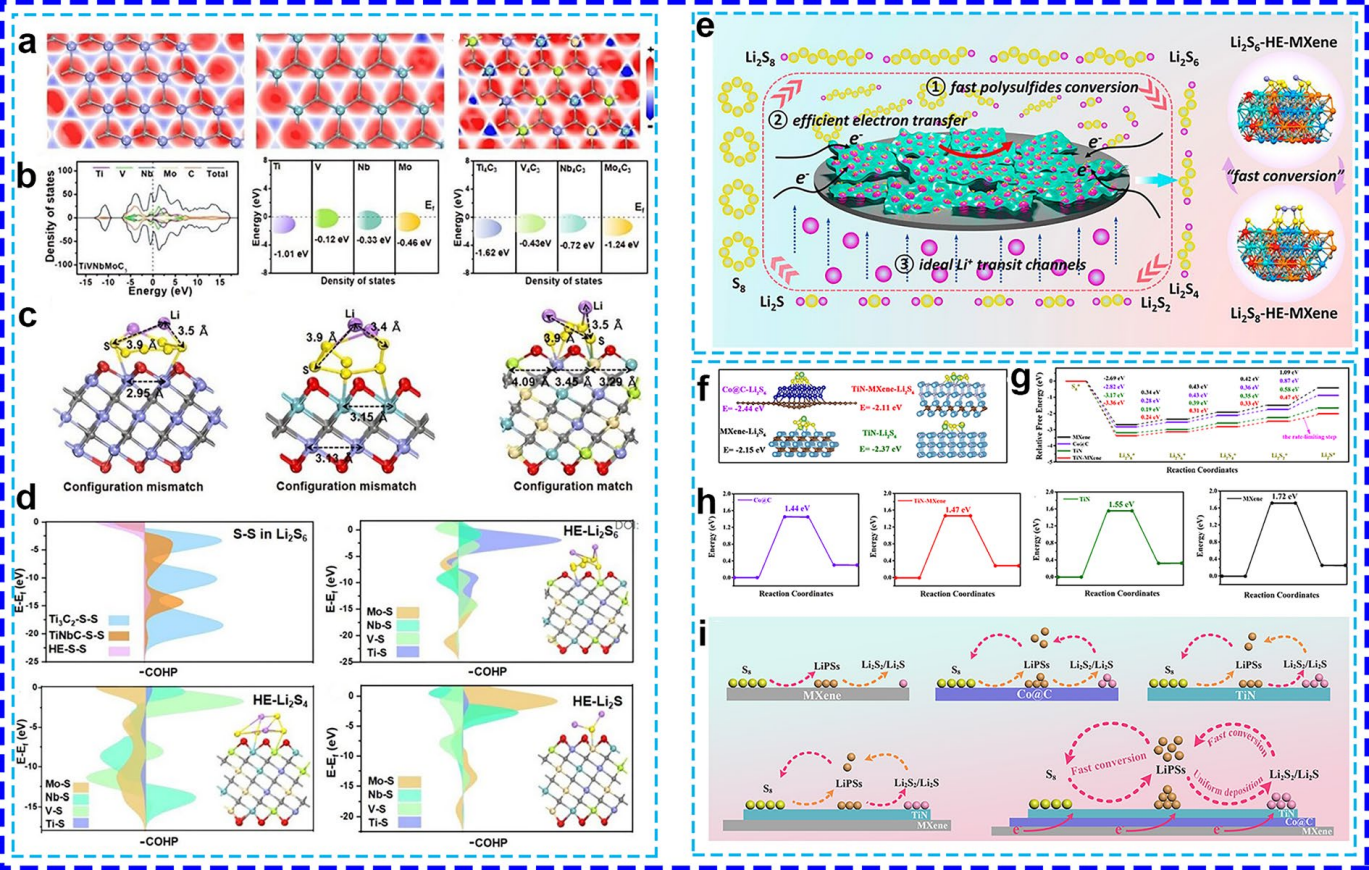
**a** Electron density, **b** DOS, **c** configurational compatibility, and **d** COHP of S–S bond in Li_2_S_6_ absorbed on various surfaces [125]. Copyright 2024, Royal Society of Chemistry. **e** Schematic illustration of the “cocktail effect” on the LiPSs conversion process [129]. Copyright 2024, American Chemical Society. **f** Binding energies, **g** ΔG profiles from S_8_ to Li_2_S, **h** Li_2_S dissociation energy barrier, and **i** catalytic mechanism of TiN-MXene-Co@CNTs for sulfur conversions [132]. Copyright 2024, WILEY–VCH

The concept of the “high entropy effect” has recently gained considerable attention in the design of electrode materials, and catalysts for electrolysis reactions [125]. Increasing the configurational entropy within materials is widely recognized for its ability to stabilize the solid solution phase, modify the electronic structure, enhance electrical conductivity, and induce lattice distortion, all of which reduce the Li^+^ diffusion barrier [126]. Furthermore, the homogeneous distribution of multiple metal elements in high entropy materials generates a “cocktail effect”, which creates numerous adsorption sites essential for catalyzing complex reactions [127]. In LSBs, this "cocktail effect" enables the various metals in high entropy materials to strongly capture LiPSs and efficiently facilitate each step of the LiPS conversion process during the overall S/Li_2_S reaction [128]. For example, a TiVNbMoC_3_ high-entropy MXene (HE-MXene), composed of four size-compatible transition metal elements uniformly distributed within the M-layer, was designed as a platform for the synergistic engineering of multi-active centers in LSBs [125]. The electron density on the HE-MXene surface was significantly increased around the variable metal sites, enhancing electron mobility and thereby lowering the reaction barrier for LSBs (Fig. 9a). Compared to Ti_3_C_2_ and TiNbC MXenes, TiVNbMoC_3_ HE-MXene exhibited a DOS closer to the Fermi level (Fig. 9b) and an extended interaction range of 3.29–4.09 Å of Li_2_S_6_ (Fig. 9c), matching well with the configurations of LiPSs. PDOS calculations revealed a substantial shift in the *d*-band center of each transition metal atom in TiVNbMoC_3_ HE-MXene towards the Fermi level. This shift was attributed to the arrangement of the four transition metal atoms in a solid-solution state, which facilitated the hybridization of Ti-3*d*, V-3*d*, Nb-3*d*, Mo-3*d*, and C-2*p* states. The M-layer engineering effectively tuned the electronic structure and *d*-band center of the MXene, resulting in a material with enhanced electron density and optimal orbital hybridization (Fig. 9d). This modification not only strengthened interactions with LiPSs but also accelerated redox reaction kinetics, thereby improving catalytic performance.

The innovative integration of high-entropy MXene and graphene has also demonstrated high electrical conductivity and provided abundant metal active sites for efficient chemisorption with LiPSs [129, 130]. Chen et al. [129] developed a high-entropy MXene-doped graphene composite (HE-MXene) as a bifunctional mediator for separator modification in LSBs. Their study demonstrated that the incorporating additional metal elements (Ti, V, and Nb) into HE-MXene enhanced continuous charge regulation and accelerated multielectron transfer. Compared to traditional transition metal carbides (TMCs) and Ti_4_C_3_, HE-MXene exhibited superior binding energies toward Li_2_S_6_ (− 20.82 eV) and Li_2_S (− 5.73 eV), as well as a significantly lower Li^+^ diffusion barrier (0.027 eV) and a reduced Li_2_S decomposition barrier (0.017 eV). These improvements are attributed to the synergistic effects of local coordination changes and charge transfer within the multi-metal quasi-atoms of HE-MXene, which promote the formation of a more stable and efficient crystal structure (Fig. 9e). Under high sulfur loading of 6.5 mg cm^−2^ and low E/S ratio of 7.1 μg mL^−1^, the cells with HE-MXene/G@PP modified separators showed outstanding capacity retention of 85.7% at 1C over 500 cycles.

In another case, a highly chaotic MXenes-based heterostructure material comprising Ti_3_C_2_T_x_ MXene sheets, TiO_2_, TiN, and TiS_2_ was designed as an efficient SRR electrocatalyst [131]. The diverse heterojunctions within the structure facilitate enhanced electron and Li^+^ transfer, thereby improving the adsorption capacity for soluble LiPSs. The combination of “high entropy”, heterostructure engineering, and MXenes significantly optimized the performance of the HCMH catalyst. This is demonstrated by a reduced Tafel slope of 62.9 mV dec^−1^ and an enhanced electron transfer number of 7.10, compared to moderately disordered samples such as TiO_2_/TiN/Ti_3_C_2_T_x_ (MCMH) and MXenes alone. DFT calculations further demonstrated that the incorporation of new phases in the HCMH structure lowered the Gibbs energy barriers for both Li_2_S_2_/Li_2_S reduction and Li_2_S decomposition. Due to its high electrical conductivity and exceptional SRR catalytic activity, the HCMH/S cell exhibited enhanced electrochemical stability, maintaining a more stable reversible capacity over extended cycling.

MXene, TiN, and Co@C each play distinct roles in the adsorption and catalytic processes of sulfur redox reactions, yet theoretical calculations indicate that no single component alone can effectively accelerate the overall sulfur redox kinetics (Fig. 9f–h) [132]. To address this, we developed the TiN-MXene-Co@CNTs composite, where Co nanoparticles were in situ grown on TiN-MXene nanosheets and encapsulated with CNTs to maximize the exposure of active sites. This composite exhibited significantly enhanced adsorption and catalytic activity for both soluble LiPSs and solid Li_2_S (Fig. 9i). The synergistic interactions between TiN, MXene, and Co@C led to a significant enhancement in cathode performance, achieving a high areal capacity of 6.3 mAh cm^−2^ under high sulfur loading of 8.9 mg cm^−2^ and a low E/S ratio. The conductive network formed by TiN-MXene-Co@CNTs as a Li host provided abundant lithiophilic sites and a high specific surface area, which in turn optimized the Li stripping/plating processes, resulting in a minimal voltage hysteresis of 13.2 mV over 1000 h. The presence of CNTs not only improved the electrical conductivity of the composite but also provided a protective layer around the Co nanoparticles, enhancing their stability. Moreover, the synergistic effect between TiN-MXene nanosheets and CNT-encapsulated Co nanoparticles facilitated the uniform distribution of Li^+^, which mitigated dendrite formation and improved the cycling stability of the battery.

The comparison of cycling performances between LSBs based on metal-based materials and MXenes@metal-based materials has demonstrated the significant potential of MXenes@metal-based materials in enhancing the cycling stability and overall performance of LSBs, as summarized in Table 2. However, current research has been predominantly focusing on Ti-based MXenes, leaving other types underexplored. Determining the optimal mass ratio of MXenes to metal-based materials is crucial for efficient LiPSs conversion and Li_2_S deposition/decomposition. In addition, it is very essential to gain a more comprehensive understanding of the structure–activity relationship between MXenes-based electrocatalysts and high-loading and lean electrolyte LSBs, which can be achieved through detailed in situ and ex situ characterizations.

**Table 2:** Comparison of cycling performances of LSBs based on metal-based materials and MXenes@metal-based materials

Samples	Sulfur loading (mg cm^−2^)	E/S ratio (μL mg^−1^)	Current density /cycling	Specific capacity (mAh g^−1^)	Area capacity (mAh cm^−2^)	Retention rate (%)	Rate performance	Ref
MXene-CNT	7.0	5.0	0.05C/80	700.0	4.90		1446(0.2C), 1225(0.5C), 1081(1.0C), 976(2.0C), 879(3.0C), 797(4.0C), 750(5.0C), 686(8.0C)	[108]
TiC_3_O_3_	7.6	–	0.1C/100	953.1	7.22	79.94	1496(0.1C), 1310(0.2C), 1140(0.5C), 978(1.0C), 748(2.0C)	[109]
P-CoSe_2_/MXene	4.0	10.0	1.0C/200	901.0	3.60	83	1469(0.1C), 1136(0.2C), 989(0.5C), 861(1.0C), 746(2.0C), 673(3.0C), 603(4.0C)	[110]
Ni-CoSe_2_/MX	1.2	–	0.2C/100	791.5	0.95	68.1	1267(0.2C), 1029(0.5C), 891(1.0C), 773(2.0C), 679(3.0C), 561(5.0C)	[114]
Co-MoSe_2_/MXenes	9.9	3.5	0.1C/50	808.1	8.00	62.2	1454(0.1C), 1390(0.2C), 1290(0.5C), 1170(1.0C), 995(2.0C), 759(5.0C)	[111]
CoD-FeP_v_@MXene	5.8	5.0	0.2C/120	821.0	4.76	98.4	1297(0.2C), 1020(0.5C), 951(1.0C), 872(2.0C), 797(3.0C), 726(4.0C)	[115]
Co-VC	7.6	4.0	0.1C/50	900.0	6.84	74.2	1212(0.2C), 1108(0.5C), 966(1.0C), 813(2.0C)	[116]
Cu SA/N-Ti_3_C_2_T_x_	7.2	–	0.1C/50	734.3	5.28	74.5	1468 (0.2C), 1193 (0.5C), 1085(1.0C), 989(2.0C), 925(3.0C)	[117]
Ru-MoS_2_/MXene	9.5	4.3	0.2C/200	726.0	6.87	88.5	1256(0.2C), 1104(0.5C), 993(1.0C), 862(2.0C) 770(3.0C), 684(6.0C)	[99]
NbB_2_-MXene	7.0	5.0	0.1C/60	928.6	6.50	80	1310(0.1C), 1097(0.2C), 902(0.5C), 782(1.0C), 678(2.0C)	[59]
SnO_2_@MXene	7.5	10.0	0.02C/50	1013.0	7.60	82.8	1231(0.1C), 1150(0.2C), 1076(0.5C), 963(1.0C), 845(2.0C)	[119]
TiO_2_-MXenes	7.3	–	0.2C/200	600.0	4.38	57.7	1374(0.1C), 1031(0.2C), 898(0.5C), 786(1.0C), 702(2.0C)	[120]
MnO_x_/MXenes	7.0	10.0	0.05C/100	887.3	5.80	55.6	1423(0.2C), 1265(0.3C), 1184(0.5C), 1063(1.0C), 936(2.0C), 817(3.0C), 709(5.0C)	[121]
SnS_2_-MXene	8.0	5.0	0.05C/50	677.8	5.43	73.9	1461(0.1C), 979(0.2C), 829(0.5C), 763(1.0C), 691(2.0C), 603(5.0C)	[11]
MXene/1 T-2H MoS_2_-C	1.0	–	0.2C/40	915.2	0.92	83.3	1194(0.1C), 1014(0.5C), 905(1.0C), 797(1.5C), 677(2.0C)	[122]
MoS_2_@Mo_2_C MXene	3.2	–	0.1C/100	755.6	2.42	92.6	1205(0.2C), 1034(0.5C), 939(1.0C), 825(2.0C), 679(3.0C), 567(5.0C)	[41]
MXene-VS_4_-SnS_2_	6.0	7.0	1.0C/100	600.0	3.60	74.4	1466(0.1C), 1323(0.2C), 1036(0.5C), 904(1.0C), 768(2.0C), 625(3.0C)	[123]
HE-MXene	5.4	8.3	0.2C/100	912.0	4.92	99.9	1238(0.2C), 880(0.5C), 736(1.0C), 638(2.0C), 545(5.0C)	[125]
HE-MXene/G	6.5	7.1	1.0C/500	520.0	3.38	69	1358(0.2C), 1191(0.4C), 1110(0.6C), 1041(0.8C), 1001(1.0C)	[129]
HCMH	5.1	6.0	0.1C/100	803.9	4.10	81.1	1466(0.1C), 1213(0.2C), 1096(0.5C), 1074(1.0C), 868(2.0C)	[131]
TiN-MXene-Co@CNTs	8.9	5.0	0.05C/50	707.8	6.30	81.4	1511(0.1C), 1251(0.2C), 1084(0.5C), 993(1.0C), 869(2.0C), 639(3.0C)	[132]
WS_2_	5	–	0.5C/300	754	6.49	86.1	1140(0.3C), 1053(0.5C), 932(1.0C), 879(2.0C), 855(3.0C)	[133]
Co_3_Mo_3_N	5.4	–	0.1C/120	776.4	3.64	84.6	1102(0.2C), 878(0.5C), 784(1.0C), 724(2.0C), 704(3.0C)	[134]
N-Co_2_VO_4_-Co	6	–	0.2C/100	701.3	6.1	6	1522(0.1C), 1406(0.2C), 1235(0.5C), 1181(1.0C), 1036(2.0C), 969(3.0C), 750(5.0C), 480(8.0C)	[135]
S@Ni-MoS_2_/rGO	5.89	–	0.2C/150	558.9	4.79	78	1152(0.1C), 944(0.2C), 844(0.5C), 786(1.0C), 757(2.0C)	[136]
TiS_2_@NSC	5.3	8	0.2C/120	745	5.6	65	–	[137]
3D P-MoS	3.7	–	0.1C/100	800	4.3	75	1099(0.1C), 1010(0.2C), 907(0.5C), 868(1.0C), 747(2.0C), 667(4.0C)	[138]
NiCo_2_S_4_	8.9	–	0.1C/70	720	6.52	78.5	1442 (0.2C), 961(0.5C), 816(1.0C), 733(2.0C), 624(3.0C)	[139]
HGCF	4.5		0.2C/100	739.8	4.9	86.3	1346 (0.1C), 983(0.2C), 887(0.5C), 814(1.0C), 742(2.0C)	[140]

## Conclusions and Perspectives

LSBs have gained significant attention as a promising technology capable of achieving high energy densities exceeding 500 Wh kg^−1^. However, reaching this target requires high-sulfur loadings and lean electrolyte conditions in the cathode. This review highlights the critical importance of the conditions in enhancing energy density, while addressing the associated challenges, including severe LiPSs diffusion, substantial volume changes during cycling, sluggish electrochemical kinetics, and increasing side reactions. Optimizing the sulfur cathode and regulating Li deposition are essential strategies to overcome these obstacles.

MXenes, a class of 2D materials with excellent conductivity, large polar surfaces, and abundant electrocatalytic active sites, have emerged as promising candidates for fabricating novel cathode structures and/or efficient electrocatalysts for LSBs. Despite their potential, the application of MXenes in high-sulfur loading and lean electrolyte LSBs is hindered by challenges such as the layer restacking, poor stability in oxygen atmospheres, and difficulties in large-scale synthesis. To address these challenges, various modification strategies have been explored, including surface optimization with more stable functional groups and structural adjustments through the manipulation of different dimensions. Additionally, the incorporation of carbonaceous materials as interlayer spacers on MXene surfaces can effectively prevent nanosheet restacking and facilitate fast charge transfer across the MXene planes. Moreover, integrating MXenes with metal-based materials such as metal oxides, sulfides, selenides, tellurides, and hydroxides allows the formation of heterostructure electrocatalysts, which create diverse catalytic sites. These sites can be further optimized for the efficient conversion of LiPSs, prevention of Li dendrite formation, and enhancement of cycling stability in LSBs. Furthermore, improvements in scalable synthesis methods, such as wet-chemical etching or electrochemical exfoliation, hold potential for overcoming production challenges of MXenes.

Despite progress in understanding the effects of MXene surface terminations and active sites on LiPSs adsorption and catalysis, the intricate interactions between MXenes and LiPSs during charge/discharge cycles are still poorly understood and difficult to observe. Future research should focus on elucidating the underlying mechanisms governing the "adsorption-diffusion-conversion" process of LiPSs and the "nucleation-decomposition" process of Li_2_S. It is also crucial to gain a deeper understanding of MXene surface chemistry, electronic structure, and reactivity toward LiPSs as well as the thermodynamics and kinetics of these processes. Key future directions include:

1. *Precise control of MXene surface terminations*: Current preparation methods for MXenes are insufficient in controlling the types, distribution, and content of surface terminations. Given that sulfur redox reactions are highly dependent on these terminations, precise control over MXene surface chemistry is essential for understanding catalytic mechanisms.
2. *Development of novel MXene species*: To date, around 30 types of MXenes have been synthesized, mostly focusing on Ti-based MXenes. Exploring new MXene compositions and terminations beyond common groups (− O, − OH, − F, − S) could significantly enhance energy density in high-loading LSBs.
3. *Real-world applications of MXenes-based electrodes*: Most experimental evaluations rely on coin-type cells with excess electrolyte and high amounts of porous carbon, conditions that do not accurately reflect real-world applications. To properly assess the performance of MXene-based electrodes, it is essential to conduct tests using pouch cells or full batteries that feature high-sulfur loading, lean electrolyte conditions, and minimal porous carbon. This more realistic setup would provide a more accurate representation of how these materials perform under practical conditions, such as those encountered in commercial LSBs.
4. *Advanced characterization technologies*: Understanding the catalytic mechanisms of MXenes-based materials is challenging due to the complexity of intermediates in LSBs during charge/discharge cycles. In situ characterization techniques like XRD, Raman, TEM, operando XAS, and UV–vis spectroscopy can track LiPS conversion processes at different stages and provide direct data for a deeper understanding of electrochemical reaction pathways.
5. *Machine learning with high-quality small datasets*: Machine learning can accelerate electrocatalyst discovery by recommending experimental conditions that converge quickly to desired properties. Active learning strategies, which optimize development using high-quality small datasets, are especially beneficial for MXenes, given the limited data available. This approach integrates known physical and chemical properties of MXenes into models to enable rapid reverse design of high-performance materials based on minimal data.

## References

[1] M. Armand, J.-M. Tarascon, Building better batteries. Nature **451**(7179), 652–657 (2008). 10.1038/451652a 18256660 10.1038/451652a

[2] L. Wang, Y. Li, Y. Ai, E. Fan, F. Zhang et al., Tracking heterogeneous interface charge reverse separation in SrTiO_3_/NiO/NiS nanofibers with *in situ* irradiation XPS. Adv. Funct. Mater. **33**(44), 2306466 (2023). 10.1002/adfm.202306466

[3] C. Xia, C.Y. Kwok, L.F. Nazar, A high-energy-density lithium–oxygen battery based on a reversible four-electron conversion to lithium oxide. Science **361**(6404), 777–781 (2018). 10.1126/science.aas9343 30139868 10.1126/science.aas9343

[4] Y. Li, L. Wang, F. Zhang, W. Zhang, G. Shao et al., Detecting and quantifying wavelength-dependent electrons transfer in heterostructure catalyst *via in situ* irradiation XPS. Adv. Sci. **10**(4), e2205020 (2023). 10.1002/advs.202205020 10.1002/advs.202205020 PMC9896054 36373728

[5] M. Winter, R.J. Brodd, What are batteries, fuel cells, and supercapacitors? Chem. Rev. **104**(10), 4245–4269 (2004). 10.1021/cr020730k 15669155 10.1021/cr020730k

[6] L. Gao, T. Sheng, M. Wang, H. Ren, S.W. Joo et al., Titanium nitride nanocrystals anchored evenly on interconnected carbon nanosheets with effective chemisorption and catalytic effects towards polysulfides for long-life lithium−sulfur batteries. Electrochim. Acta **395**, 139208 (2021). 10.1016/j.electacta.2021.139208

[7] X. Pang, H. Geng, S. Dong, B. An, S. Zheng et al., Medium-entropy-alloy FeCoNi enables lithium–sulfur batteries with superb low-temperature performance. Small **19**(5), e2205525 (2023). 10.1002/smll.202205525 36433827 10.1002/smll.202205525

[8] W. Yan, J.-L. Yang, X. Xiong, L. Fu, Y. Chen et al., Versatile asymmetric separator with dendrite-free alloy anode enables high-performance Li–S batteries. Adv. Sci. **9**(25), e2202204 (2022). 10.1002/advs.202202204 10.1002/advs.202202204 PMC9443453 35748192

[9] W. Liu, C. Luo, S. Zhang, B. Zhang, J. Ma et al., Cobalt-doping of molybdenum disulfide for enhanced catalytic polysulfide conversion in lithium–sulfur batteries. ACS Nano **15**(4), 7491–7499 (2021). 10.1021/acsnano.1c00896 33834767 10.1021/acsnano.1c00896

[10] S. Fu, H. Wang, S. Schaefer, B. Shang, L. Ren et al., Simple framework for simultaneous analysis of both electrodes in stoichiometric lithium–sulfur batteries. J. Am. Chem. Soc. **146**(31), 21721–21728 (2024). 10.1021/jacs.4c05827 39051979 10.1021/jacs.4c05827

[11] L. Chen, L. Yue, X. Wang, S. Wu, W. Wang et al., Synergistically accelerating adsorption-electrocataysis of sulfur species *via* interfacial built-In electric field of SnS_2_-MXene Mott-Schottky heterojunction in Li–S batteries. Small **19**(15), 2206462 (2023). 10.1002/smll.202206462 10.1002/smll.202206462 36642788

[12] Y. Li, Y. Zhang, R. Hou, Y. Ai, M. Cai et al., Revealing electron numbers-binding energy relationships in heterojunctions *via in situ* irradiated XPS. Appl. Catal. B Environ. Energy **356**, 124223 (2024). 10.1016/j.apcatb.2024.124223

[13] C.Q. Zhang, J.J. Biendicho, T. Zhang, R.F. Du, J.S. Li et al., Combined high catalytic activity and efficient polar tubular nanostructure in urchin-like metallic NiCo_2_Se_4_ for high-performance lithium–sulfur batteries. Adv. Funct. Mater. **29**(34), 1903842 (2019). 10.1002/adfm.201903842

[14] Z.X. Shi, Y.F. Ding, Q. Zhang, J.Y. Sun, Electrocatalyst modulation toward bidirectional sulfur redox in Li–S batteries: from strategic probing to mechanistic understanding. Adv. Energy Mater. **12**(29), 2201056 (2022). 10.1002/aenm.202201056

[15] P. Zhang, Y. Zhao, Y. Li, N. Li, S. Ravi, P. Silva et al., Revealing the selective bifunctional electrocatalytic sites *via in situ* irradiated X-ray photoelectron spectroscopy for lithium–sulfur battery. Adv. Sci. **10**(8), e2206786 (2023). 10.1002/advs.202206786 10.1002/advs.202206786 PMC10015878 36646512

[16] Z. Shi, S. Thomas et al., Solvation sheath reorganization by alkyl chain tuning promises lean-electrolyte Li–S batteries. ACS Energy Lett. **9**(11), 5391–5402 (2024). 10.1021/acsenergylett.4c02049

[17] Z. Shi, Z. Tian, D. Guo, Y. Wang, Z. Bayhan et al., Kinetically favorable Li–S battery electrolytes. ACS Energy Lett. **8**(7), 3054–3080 (2023). 10.1021/acsenergylett.3c00826

[18] J. Li, L. Gao, F. Pan, C. Gong, L. Sun et al., Engineering strategies for suppressing the shuttle effect in lithium–sulfur batteries. Nano-Micro Lett. **16**(1), 12 (2023). 10.1007/s40820-023-01223-1 10.1007/s40820-023-01223-1 PMC10638349 37947874

[19] Y. Chen, T. Wang, H. Tian, D. Su, Q. Zhang et al., Advances in lithium–sulfur batteries: from academic research to commercial viability. Adv. Mater. **33**(29), e2003666 (2021). 10.1002/adma.202003666 34096100 10.1002/adma.202003666

[20] A. Bhargav, J. He, A. Gupta, A. Manthiram, Lithium–sulfur batteries: attaining the critical metrics. Joule **4**(2), 285–291 (2020). 10.1016/j.joule.2020.01.001

[21] M. Zhao, B.-Q. Li, H.-J. Peng, H. Yuan, J.-Y. Wei et al., Lithium–sulfur batteries under lean electrolyte conditions: challenges and opportunities. Angew. Chem. Int. Ed. **59**(31), 12636–12652 (2020). 10.1002/anie.201909339 10.1002/anie.201909339 31490599

[22] Y.-C. Ho, S.-H. Chung, A design of the cathode substrate for high-loading polysulfide cathodes in lean-electrolyte lithium–sulfur cells. Chem. Eng. J. **422**, 130363 (2021). 10.1016/j.cej.2021.130363

[23] L.P. Chen, Y.H. Xu, G.Q. Cao, H.M.K. Sari, R.X. Duan et al., Bifunctional catalytic effect of CoSe_2_ for lithium–sulfur batteries: single doping versus dual doping. Adv. Funct. Mater. **32**(8), 2107838 (2022). 10.1002/adfm.202107838

[24] Z.W. Seh, Y. Sun, Q. Zhang, Y. Cui, Designing high-energy lithium–sulfur batteries. Chem. Soc. Rev. **45**(20), 5605–5634 (2016). 10.1039/c5cs00410a 27460222 10.1039/c5cs00410a

[25] M. Zhao, B.-Q. Li, X. Chen, J. Xie, H. Yuan et al., Redox comediation with organopolysulfides in working lithium–sulfur batteries. Chem **6**(12), 3297–3311 (2020). 10.1016/j.chempr.2020.09.015

[26] E. Kim, S. Kim, H.M. Jin, G. Kim, H.H. Ha et al., Unlocking novel functionality: pseudocapacitive sensing in MXene-based flexible supercapacitors. Nano-Micro Lett. **17**, 86 (2025). 10.1007/s40820-024-01567-2 10.1007/s40820-024-01567-2 PMC11628472 39652269

[27] I. Hussain, A. Hanan, F. Bibi, O.J. Kewate, M.S. Javed et al., Non-Ti (M_2_X and M_3_X_2_) MXenes for energy storage/conversion. Adv. Energy Mater. **14**(34), 2401650 (2024). 10.1002/aenm.202401650

[28] R. Zhao, C. Liu, Y. Zhu, G. Zou, H. Hou et al., Pathways for MXenes in solving the issues of zinc-ion batteries: achievements and perspectives. Adv. Funct. Mater. **34**(28), 2316643 (2024). 10.1002/adfm.202316643

[29] H. Zhang, C. Hao, T. Fu, D. Yu, J. Howe et al., Gradient-layered MXene/hollow lignin nanospheres architecture design for flexible and stretchable supercapacitors. Nano-Micro Lett. **17**(1), 43 (2024). 10.1007/s40820-024-01512-3 39417914 10.1007/s40820-024-01512-3 PMC11486903

[30] X. Zhong, D. Wang, J. Sheng, Z. Han, C. Sun et al., Freestanding and sandwich MXene-based cathode with suppressed lithium polysulfides shuttle for flexible lithium–sulfur batteries. Nano Lett. **22**(3), 1207–1216 (2022). 10.1021/acs.nanolett.1c04377 35084869 10.1021/acs.nanolett.1c04377

[31] X. Huang, J. Tang, T. Qiu, R. Knibbe, Y. Hu et al., Nanoconfined topochemical conversion from MXene to ultrathin non-layered TiN nanomesh toward superior electrocatalysts for lithium–sulfur batteries. Small **17**(32), e2101360 (2021). 10.1002/smll.202101360 34216427 10.1002/smll.202101360

[32] D. Zhang, S. Wang, R.M. Hu, J.N. Gu, Y.L.S. Cui et al., Catalytic conversion of polysulfides on single atom zinc implanted MXene toward high-rate lithium–sulfur batteries. Adv. Funct. Mater. **30**(30), 2002471 (2020). 10.1002/adfm.202002471

[33] M.Q. Long, K.K. Tang, J. Xiao, J.Y. Li, J. Chen et al., Recent advances on MXene based materials for energy storage applications. Mater. Today Sustain. **19**, 100163 (2022). 10.1016/j.mtsust.2022.100163

[34] P. Wang, T. Xu, B. Xi, J. Yuan, N. Song et al., A Zn_8_ double-cavity metallacalix[8]arene as molecular sieve to realize self-cleaning intramolecular tandem transformation of Li−S chemistry. Adv. Mater. **34**, 2207689 (2022). 10.1002/adma.202207689 10.1002/adma.202207689 36259588

[35] X. Zhu, T. Bian, X. Song, M. Zheng, Z. Shen et al., Accelerating S↔Li_2_S reactions in Li-S batteries through activation of S/Li_2_S with a bifunctional semiquinone catalyst. Angew. Chem. Int. Ed. **63**(5), e202315087 (2024). 10.1002/anie.202315087 10.1002/anie.202315087 38087471

[36] L. Zhou, X. Zhang, W. Hao, S. Sun, R. Wang et al., Mirror plane effect of magnetoplumbite-type oxide restraining long-chain polysulfides disproportionation for high loading lithium sulfur batteries. Small Meth. **8**(12), 2400475 (2024). 10.1002/smtd.202400475 10.1002/smtd.202400475 38837890

[37] Y. He, X. Jing, T. Lai, D. Jiang, C. Wan et al., Amphipathic emulsion binder for enhanced performance of lithium–sulfur batteries. J. Mater. Chem. A **12**(21), 12681–12690 (2024). 10.1039/d4ta01037j

[38] R. Hou, S. Zhang, Y. Zhang, N. Li, S. Wang et al., A “three-region” configuration for enhanced electrochemical kinetics and high-areal capacity lithium–sulfur batteries. Adv. Funct. Mater. **32**(19), 2200302 (2022). 10.1002/adfm.202200302

[39] F. Zhou, Y. Mei, Q. Wu, H. Li, J. Xu et al., Sulfur electrode tolerance and polysulfide conversion promoted by the supramolecular binder with rare-earth catalysis in lithium–sulfur batteries. Energy Storage Mater. **67**, 103315 (2024). 10.1016/j.ensm.2024.103315

[40] H. Xu, Q. Jiang, K.S. Hui, S. Wang, L. Liu et al., Interfacial “double-terminal binding sites” catalysts synergistically boosting the electrocatalytic Li_2_S redox for durable lithium–sulfur batteries. ACS Nano **18**(12), 8839–8852 (2024). 10.1021/acsnano.3c11903 38465917 10.1021/acsnano.3c11903 PMC10976959

[41] Y. Li, Y. Zuo, X. Li, Y. Zhang, C. Ma et al., Electron delocalization-enhanced sulfur reduction kinetics on an MXene-derived heterostructured electrocatalyst. Nano Res. **17**(8), 7153–7162 (2024). 10.1007/s12274-024-6682-6

[42] J. Feng, C. Shi, X. Zhao, Y. Zhang, S. Chen et al., Physical field effects to suppress polysulfide shuttling in lithium–sulfur battery. Adv. Mater. **36**(48), 2414047 (2024). 10.1002/adma.202414047 10.1002/adma.202414047 39402772

[43] H. Pan, Z. Cheng, Z. Zhou, S. Xie, W. Zhang et al., Boosting lean electrolyte lithium–sulfur battery performance with transition metals: a comprehensive review. Nano-Micro Lett. **15**(1), 165 (2023). 10.1007/s40820-023-01137-y 10.1007/s40820-023-01137-y PMC10310691 37386313

[44] P. Wang, F. Sun, S. Xiong, Z. Zhang, B. Duan et al., WSe_2_ flakelets on N-doped graphene for accelerating polysulfide redox and regulating Li plating. Angew. Chem. Int. Ed. **61**(7), e202116048 (2022). 10.1002/anie.202116048 10.1002/anie.202116048 34889508

[45] T. Ma, J. Deng, Y. Lin, Q. Liang, L. Hu et al., Li-rich organosulfur cathode with boosted kinetics for high-energy lithium–sulfur batteries. Energy Environ. Mater. **7**(4), e12704 (2024). 10.1002/eem2.12704

[46] Q. Wu, K. Chen, Z. Shadike, C. Li, Relay-type catalysis by a dual-metal single-atom system in a waste biomass derivative host for high-rate and durable Li–S batteries. ACS Nano **18**(21), 13468–13483 (2024). 10.1021/acsnano.3c09919 38739894 10.1021/acsnano.3c09919

[47] Z. Shi, Z. Sun, J. Cai, X. Yang, C. Wei et al., Manipulating electrocatalytic Li_2_S redox *via* selective dual-defect engineering for Li–S batteries. Adv. Mater. **33**(43), e2103050 (2021). 10.1002/adma.202103050 34463382 10.1002/adma.202103050

[48] J. Feng, C. Shi, X. Zhao, Y. Zhang, S. Chen et al., Physical field effects to suppress polysulfide shuttling in lithium–sulfur battery. Adv. Mater. **36**(48), e2414047 (2024). 10.1002/adma.202414047 39402772 10.1002/adma.202414047

[49] Z.X. Shi, M. Li, J.Y. Sun, Z.W. Chen, Defect engineering for expediting Li–S chemistry: strategies, mechanisms, and perspectives. Adv. Energy Mater. **11**(23), 2100332 (2021). 10.1002/aenm.202100332

[50] Z. Ye, Y. Jiang, T. Yang, L. Li, F. Wu et al., Engineering catalytic CoSe–ZnSe heterojunctions anchored on graphene aerogels for bidirectional sulfur conversion reactions. Adv. Sci. **9**(1), e2103456 (2022). 10.1002/advs.202103456 10.1002/advs.202103456 PMC8728854 34708583

[51] B. Li, T. Zhang, Z. Song, W. Jiang, J. Yang et al., 3D adsorption-mediator network polymer binders improve redox kinetics and flame retardant performance for high loading lithium–sulfur batteries. Adv. Funct. Mater. **33**(52), 2306990 (2023). 10.1002/adfm.202306990

[52] Y. Zhang, Z. Wu, S. Wang, Complex permittivity-dependent plasma confinement-assisted growth of asymmetric vertical graphene nanofiber membrane for high-performance Li–S full cells. InfoMat **4**(7), e12294 (2022). 10.1002/inf2.12294

[53] Z. Xu, Y. Ren, X. Shen, K. Yao, J. Li et al., PTFE nanofiber cross-linked acetylene black: a flexible self-supporting semi-confined architecture for ultra-high sulfur loading and areal capacity. Energy Storage Mater. **64**, 103071 (2024). 10.1016/j.ensm.2023.103071

[54] L. Wang, X. Meng, X. Wang, M. Zhen, Dual-conductive CoSe_2_@TiSe_2_-C heterostructures promoting overall sulfur redox kinetics under high sulfur loading and lean electrolyte. Small **19**(21), 2300089 (2023). 10.1002/smll.202300089 10.1002/smll.202300089 36843272

[55] H. Zhu, S. Chen, X. Yao, K. Yang, W. Zhao et al., Upcycling spent cathode materials to bifunctional catalysts for high-stability lithium–sulfur batteries. Adv. Funct. Mater. **34**(29), 2401470 (2024). 10.1002/adfm.202401470

[56] D. Yang, J. Wang, C. Lou, M. Li, C. Zhang et al., Single-atom catalysts with unsaturated Co–N_2_ active sites based on a C_2_N 2D-organic framework for efficient sulfur redox reaction. ACS Energy Lett. **9**(5), 2083–2091 (2024). 10.1021/acsenergylett.4c00771

[57] T.A. Oyehan, B.A. Salami, A.A. Abdulrasheed, H.U. Hambali, A. Gbadamosi et al., MXenes: Synthesis, properties, and applications for sustainable energy and environment. Appl. Mater. Today **35**, 101993 (2023). 10.1016/j.apmt.2023.101993

[58] W.Y. Lieu, C.J. Lin, X.L. Li, S.Q. Jiang, Y.J. Li et al., Structural design of electrocatalyst-decorated MXenes on sulfur spheres for lithium–sulfur batteries. Nano Lett. **23**, 5762–5769 (2023). 10.1021/acs.nanolett.3c01558 37310729 10.1021/acs.nanolett.3c01558

[59] D. Lu, X. Wang, Y. Hu, L. Yue, Z. Shao et al., Expediting stepwise sulfur conversion *via* spontaneous built-In electric field and binary sulfiphilic effect of conductive NbB_2_-MXene heterostructure in lithium–sulfur batteries. Adv. Funct. Mater. **33**(15), 2212689 (2023). 10.1002/adfm.202212689

[60] C. Jiao, C.-R. Zhao, L. Zhang, S.-Q. Zhao, G.-Y. Pang et al., Electrochemical properties of high-loading sulfur–carbon materials prepared by *in situ* generation method. Rare Met. **42**(11), 3877–3885 (2023). 10.1007/s12598-019-01262-x

[61] Z. Liu, M. Chen, D. Zhou, Z. Xiao, Scavenging of “dead sulfur” and “dead lithium” revealed by integrated–heterogeneous catalysis for advanced lithium–sulfur batteries. Adv. Funct. Mater. **33**(46), 2306321 (2023). 10.1002/adfm.202306321

[62] R. Li, L. Yang, L. Song, C. Zhou, J. Zhou et al., A thin LiGa alloy layer from *in situ* electroreduction to suppress anode dendrite formation in lithium–sulfur pouch cell. Chem. Eng. J. **455**, 140707 (2023). 10.1016/j.cej.2022.140707

[63] N. Li, L. Yu, J. Xi, Integrated design of interlayer/current-collector: heteronanowires decorated carbon microtube fabric for high-loading and lean-electrolyte lithium–sulfur batteries. Small **17**(37), 2103001 (2021). 10.1002/smll.202103001 10.1002/smll.202103001 34331399

[64] Q. Gao, Z. Shen, Z. Guo, M. Li, J. Wei et al., Metal coordinated polymer as three-dimensional network binder for high sulfur loading cathode of lithium–sulfur battery. Small **19**(28), e2301344 (2023). 10.1002/smll.202301344 36971297 10.1002/smll.202301344

[65] R. Li, Y. Zeng, L. Song, J. Lv, C. Wang et al., Mechanism and solution of overcharge effect in lithium–sulfur batteries. Small **20**(2), 2305283 (2024). 10.1002/smll.202305283 10.1002/smll.202305283 37661577

[66] L. Liang, L. Niu, T. Wu, D. Zhou, Z. Xiao, Fluorine-free fabrication of MXene *via* photo-Fenton approach for advanced lithium-sulfur batteries. ACS Nano **16**(5), 7971–7981 (2022). 10.1021/acsnano.2c00779 35466669 10.1021/acsnano.2c00779

[67] X. Liang, A. Garsuch, L.F. Nazar, Sulfur cathodes based on conductive MXene nanosheets for high-performance lithium–sulfur batteries. Angew. Chem. Int. Ed. **54**(13), 3907–3911 (2015). 10.1002/anie.201410174 10.1002/anie.201410174 25650042

[68] H. Tang, W. Li, L. Pan, K. Tu, F. Du et al., A robust, freestanding MXene-sulfur conductive paper for long-lifetime Li–S batteries. Adv. Funct. Mater. **29**(30), 1901907 (2019). 10.1002/adfm.201901907

[69] H. Tang, W.L. Li, L.M. Pan, C.P. Cullen, Y. Liu et al., *In situ* formed protective barrier enabled by Sulfur@Titanium carbide (MXene) ink for achieving high-capacity, long lifetime Li-S batteries. Adv. Sci. **5**(9), 1800502 (2018). 10.1002/advs.201800502 10.1002/advs.201800502 PMC6145260 30250792

[70] N. Li, Y. Xie, S. Peng, X. Xiong, K. Han, Ultra-lightweight Ti_3_C_2_T_x_ MXene modified separator for Li–S batteries: thickness regulation enabled polysulfide inhibition and lithium ion transportation. J. Energy Chem. **42**, 116–125 (2020). 10.1016/j.jechem.2019.06.014

[71] G. Valurouthu, M. Shekhirev, M. Anayee, R.J. Wang, K. Matthews et al., Screening conductive MXenes for lithium polysulfide adsorption. Adv. Funct. Mater. **34**(45), 2404430 (2024). 10.1002/adfm.202404430

[72] C.L. Wei, Y. Tao, Y.L. An, Y. Tian, Y.C. Zhang et al., Recent advances of emerging 2D MXene for stable and dendrite-free metal anodes. Adv. Funct. Mater. **30**(45), 2004613 (2020). 10.1002/adfm.202004613

[73] D. Zhang, S. Wang, B. Li, Y. Gong, S. Yang, Horizontal growth of lithium on parallelly aligned MXene layers towards dendrite-free metallic lithium anodes. Adv. Mater. **31**(33), e1901820 (2019). 10.1002/adma.201901820 31231876 10.1002/adma.201901820

[74] D. Wang, F. Li, R. Lian, J. Xu, D. Kan et al., A general atomic surface modification strategy for improving anchoring and electrocatalysis behavior of Ti_3_C_2_T_2_ MXene in lithium–sulfur batteries. ACS Nano **13**(10), 11078–11086 (2019). 10.1021/acsnano.9b03412 31469546 10.1021/acsnano.9b03412

[75] Y. Li, Y.-C. Zhu, S. Vallem, M. Li, S. Song et al., Flame-retardant ammonium polyphosphate/MXene decorated carbon foam materials as polysulfide traps for fire-safe and stable lithium–sulfur batteries. J. Energy Chem. **89**, 313–323 (2024). 10.1016/j.jechem.2023.10.029

[76] B. Li, P. Wang, J. Yuan, N. Song, J. Feng et al., P-doped RuSe_2_ on porous N-doped carbon matrix as catalysts for accelerated sulfur redox reactions. Angew. Chem. Int. Ed. **63**(48), e202408906 (2024). 10.1002/anie.202408906 10.1002/anie.202408906 39196702

[77] W. Yu, S. Ma, M. He, R. Li, H. Yang et al., Immobilization and kinetic acceleration of lithium polysulfides by iodine-doped MXene nanosheets in lithium–sulfur batteries. J. Phys. Chem. C **126**(27), 10986–10994 (2022). 10.1021/acs.jpcc.2c02689

[78] J. Feng, W. Liu, C. Shi, C. Zhang, X. Zhao et al., Enabling fast diffusion/conversion kinetics by thiourea-induced wrinkled N, S Co-doped functional MXene for lithium–sulfur battery. Energy Storage Mater. **67**, 103328 (2024). 10.1016/j.ensm.2024.103328

[79] W. Zhang, H. Jin, Y. Du, G. Chen, J. Zhang, Sulfur and nitrogen codoped Nb_2_C MXene for dendrite-free lithium metal battery. Electrochim. Acta **390**, 138812 (2021). 10.1016/j.electacta.2021.138812

[80] P. Li, H. Lv, Z. Li, X. Meng, Z. Lin et al., The electrostatic attraction and catalytic effect enabled by ionic-covalent organic nanosheets on MXene for separator modification of lithium–sulfur batteries. Adv. Mater. **33**(17), e2007803 (2021). 10.1002/adma.202007803 33734507 10.1002/adma.202007803

[81] Y. Cao, Y. Jia, X. Meng, X. Fan, J. Zhang et al., Covalently grafting conjugated porous polymers to MXene offers a two-dimensional sandwich-structured electrocatalytic sulfur host for lithium–sulfur batteries. Chem. Eng. J. **446**, 137365 (2022). 10.1016/j.cej.2022.137365

[82] X. Wang, C. Yang, X. Xiong, G. Chen, M. Huang et al., A robust sulfur host with dual lithium polysulfide immobilization mechanism for long cycle life and high capacity Li–S batteries. Energy Storage Mater. **16**, 344–353 (2019). 10.1016/j.ensm.2018.06.015

[83] X. Li, Q. Guan, Z. Zhuang, Y. Zhang, Y. Lin et al., Ordered mesoporous carbon grafted MXene catalytic heterostructure as Li-ion kinetic pump toward high-efficient sulfur/sulfide conversions for Li–S battery. ACS Nano **17**(2), 1653–1662 (2023). 10.1021/acsnano.2c11663 10.1021/acsnano.2c11663 36607402

[84] C. Wei, Y. Wang, Y. Zhang, L. Tan, Y. Qian et al., Flexible and stable 3D lithium metal anodes based on self-standing MXene/COF frameworks for high-performance lithium–sulfur batteries. Nano Res. **14**(10), 3576–3584 (2021). 10.1007/s12274-021-3433-9

[85] C. Wen, X. Zheng, X. Li, M. Yuan, H. Li et al., Rational design of 3D hierarchical MXene@AlF_3_/Ni(OH)_2_ nanohybrid for high-performance lithium–sulfur batteries. Chem. Eng. J. **409**, 128102 (2021). 10.1016/j.cej.2020.128102

[86] D. Xiong, S. Huang, D. Fang, D. Yan, G. Li et al., Porosity engineering of MXene membrane towards polysulfide inhibition and fast lithium ion transportation for lithium–sulfur batteries. Small **17**(34), e2007442 (2021). 10.1002/smll.202007442 34278712 10.1002/smll.202007442

[87] W. Zhao, Y. Lei, Y. Zhu, Q. Wang, F. Zhang et al., Hierarchically structured Ti_3_C_2_T_x_ MXene paper for Li-S batteries with high volumetric capacity. Nano Energy **86**, 106120 (2021). 10.1016/j.nanoen.2021.106120

[88] Z. Xiao, Z. Li, P. Li, X. Meng, R. Wang, Ultrafine Ti_3_C_2_ MXene nanodots-interspersed nanosheet for high-energy-density lithium–sulfur batteries. ACS Nano **13**(3), 3608–3617 (2019). 10.1021/acsnano.9b00177 30864777 10.1021/acsnano.9b00177

[89] C. Wei, M. Tian, Z. Fan, L. Yu, Y. Song et al., Concurrent realization of dendrite-free anode and high-loading cathode *via* 3D printed N–Ti_3_C_2_ MXene framework toward advanced Li–S full batteries. Energy Storage Mater. **41**, 141–151 (2021). 10.1016/j.ensm.2021.05.030

[90] H.J. Peng, G. Zhang, X. Chen, Z.-W. Zhang, W.T. Xu et al., Enhanced electrochemical kinetics on conductive polar mediators for lithium–sulfur batteries. Angew. Chem. Int. Ed. **55**, 12990 (2016). 10.1002/anie.201605676 10.1002/anie.201605676 27513988

[91] P.-Y. Zhai, J.-Q. Huang, L. Zhu, J.-L. Shi, W. Zhu et al., Calendering of free-standing electrode for lithium–sulfur batteries with high volumetric energy density. Carbon **111**, 493–501 (2017). 10.1016/j.carbon.2016.10.035

[92] C. Lin, C. Niu, X. Xu, K. Li, Z. Cai et al., A facile synthesis of three dimensional graphene sponge composited with sulfur nanoparticles for flexible Li–S cathodes. Phys. Chem. Chem. Phys. **18**(32), 22146–22153 (2016). 10.1039/c6cp03624d 27443983 10.1039/c6cp03624d

[93] Z. Yuan, H.-J. Peng, J.-Q. Huang, X.-Y. Liu, D.-W. Wang et al., Hierarchical free-standing carbon-nanotube paper electrodes with ultrahigh sulfur-loading for lithium–sulfur batteries. Adv. Funct. Mater. **24**(39), 6105–6112 (2014). 10.1002/adfm.201401501

[94] X. Liang, Y. Rangom, C.Y. Kwok, Q. Pang, L.F. Nazar, Interwoven MXene nanosheet/carbon-nanotube composites as Li-S cathode hosts. Adv. Mater. **29**(3), 1603040 (2017). 10.1002/adma.201603040 10.1002/adma.201603040 27859697

[95] W. Bao, L. Liu, C. Wang, S. Choi, D. Wang et al., Facile synthesis of crumpled nitrogen-doped MXene nanosheets as a new sulfur host for lithium–sulfur batteries. Adv. Energy Mater. **8**(13), 1702485 (2018). 10.1002/aenm.201702485

[96] L. Zhu, H.-J. Peng, J. Liang, J.-Q. Huang, C.-M. Chen et al., Interconnected carbon nanotube/graphene nanosphere scaffolds as free-standing paper electrode for high-rate and ultra-stable lithium–sulfur batteries. Nano Energy **11**, 746–755 (2015). 10.1016/j.nanoen.2014.11.062

[97] J. Li, L. Liu, J. Wang, Y. Zhuang, B. Wang et al., Freestanding TiO_2_ nanoparticle-embedded high directional carbon composite host for high-loading low-temperature lithium–sulfur batteries. ACS Sustain. Chem. Eng. **11**(9), 3657–3663 (2023). 10.1021/acssuschemeng.2c06482

[98] H. Li, P. Shi, L. Wang, T. Yan, T. Guo et al., Cooperative catalysis of polysulfides in lithium–sulfur batteries through adsorption competition by tuning cationic geometric configuration of dual-active sites in spinel oxides. Angew. Chem. Int. Ed. **62**(8), e202216286 (2023). 10.1002/anie.202216286 10.1002/anie.202216286 36546717

[99] Y. Bai, T.T. Nguyen, H. Song, R. Chu, D.T. Tran et al., Ru single atom dispersed on MoS_2_/MXene for enhanced sulfur reduction reaction in lithium–sulfur batteries. Small **20**(38), e2402074 (2024). 10.1002/smll.202402074 38794990 10.1002/smll.202402074

[100] M. Fang, J. Han, S. He, J.-C. Ren, S. Li et al., Effective screening descriptor for MXenes to enhance sulfur reduction in lithium-sulfur batteries. J. Am. Chem. Soc. **145**(23), 12601–12608 (2023). 10.1021/jacs.3c01834 37276342 10.1021/jacs.3c01834

[101] Q. Zeng, L. Xu, G. Li, Q. Zhang, S. Guo et al., Integrating sub-nano catalysts into metal-organic framework toward pore-confined polysulfides conversion in lithium–sulfur batteries. Adv. Funct. Mater. **33**(43), 2304619 (2023). 10.1002/adfm.202304619

[102] Y. Zhang, C. Kang, W. Zhao, Y. Song, J. Zhu et al., D-p hybridization-induced “trapping-coupling-conversion” enables high-efficiency Nb single-atom catalysis for Li–S batteries. J. Am. Chem. Soc. **145**(3), 1728–1739 (2023). 10.1021/jacs.2c10345 36640116 10.1021/jacs.2c10345

[103] Y. Guo, X. Yang, X. Liu, X. Tong, N. Yang, Coupling methanol oxidation with hydrogen evolution on bifunctional Co-doped Rh electrocatalyst for efficient hydrogen generation. Adv. Funct. Mater. **33**(2), 2209134 (2023). 10.1002/adfm.202209134

[104] Z. Hou, C. Cui, Y. Li, Y. Gao, D. Zhu et al., Lattice-strain engineering for heterogenous electrocatalytic oxygen evolution reaction. Adv. Mater. **35**(39), 2209876 (2023). 10.1002/adma.202209876 10.1002/adma.202209876 36639855

[105] Z. Shen, X. Jin, J. Tian, M. Li, Y. Yuan et al., Cation-doped ZnS catalysts for polysulfide conversion in lithium–sulfur batteries. Nat. Catal. **5**(6), 555–563 (2022). 10.1038/s41929-022-00804-4

[106] X.-L. Zhang, S.-J. Hu, Y.-R. Zheng, R. Wu, F.-Y. Gao et al., Polymorphic cobalt diselenide as extremely stable electrocatalyst in acidic media *via* a phase-mixing strategy. Nat. Commun. **10**(1), 5338 (2019). 10.1038/s41467-019-12992-y 31767845 10.1038/s41467-019-12992-y PMC6877578

[107] X.-L. Zhang, P.-C. Yu, X.-Z. Su, S.-J. Hu, L. Shi et al., Efficient acidic hydrogen evolution in proton exchange membrane electrolyzers over a sulfur-doped marcasite-type electrocatalyst. Sci. Adv. **9**(27), eadh2885 (2023). 10.1126/sciadv.adh2885 37406120 10.1126/sciadv.adh2885 PMC10321749

[108] X. Wang, D. Luo, J. Wang, Z. Sun, G. Cui et al., Strain engineering of a MXene/CNT hierarchical porous hollow microsphere electrocatalyst for a high-efficiency lithium polysulfide conversion process. Angew. Chem. Int. Ed. **60**(5), 2371–2378 (2021). 10.1002/anie.202011493 10.1002/anie.202011493 33398902

[109] C. Zhang, W. Chu, X. Hong, Q. He, R. Lu et al., Accelerating conversion of LiPSs on strain-induced MXene for high-performance Li–S battery. Chem. Eng. J. **439**, 135679 (2022). 10.1016/j.cej.2022.135679

[110] J. Wang, Y. Xu, Y. Zhuang, Y. Li, H.H. Chang et al., Lattice strain and charge localization dual regulation of phosphorus-doped CoSe_2_/MXene catalysts enable kinetics-enhanced and dendrite-free lithium–sulfur batteries. Adv. Energy Mater. **14**(38), 2401630 (2024). 10.1002/aenm.202401630

[111] W. Wang, L. Huai, S. Wu, J. Shan, J. Zhu et al., Ultrahigh-volumetric-energy-density lithium–sulfur batteries with lean electrolyte enabled by cobalt-doped MoSe_2_/Ti_3_C_2_T_*x*_ MXene bifunctional catalyst. ACS Nano **15**(7), 11619–11633 (2021). 10.1021/acsnano.1c02047 34247479 10.1021/acsnano.1c02047

[112] S. Hu, T. Wang, B. Lu, D. Wu, H. Wang et al., Ionic-liquid-assisted synthesis of FeSe–MnSe heterointerfaces with abundant Se vacancies embedded in N, B Co-doped hollow carbon microspheres for accelerating the sulfur reduction reaction. Adv. Mater. **34**(41), e2204147 (2022). 10.1002/adma.202204147 35900291 10.1002/adma.202204147

[113] K. Xu, X. Liu, J. Liang, J. Cai, K. Zhang et al., Manipulating the redox kinetics of Li–S chemistry by tellurium doping for improved Li–S batteries. ACS Energy Lett. **3**(2), 420–427 (2018). 10.1021/acsenergylett.7b01249

[114] Z. Wang, H. Jiang, Z. Ni, C. Wei, K. Tian et al., Spatial confinement design with metal-doped catalysts: modulating electronic-state of active sites for accelerating sulfur redox kinetics in lithium–sulfur batteries. Adv. Funct. Mater. **35**, 2416997 (2025). 10.1002/adfm.202416997

[115] X. Zhou, Y. Cui, X. Huang, X. Wu, H. Sun et al., Dual-defect engineering of bidirectional catalyst for high-performing lithium–sulfur batteries. Small **19**(40), e2301545 (2023). 10.1002/smll.202301545 37287408 10.1002/smll.202301545

[116] Y. Song, Y. Sun, L. Chen, L. Song, Q. Yang et al., Seeding Co atoms on size effect-enabled V_2_C MXene for kinetically boosted lithium–sulfur batteries. Adv. Funct. Mater. **34**(51), 2409748 (2024). 10.1002/adfm.202409748

[117] H. Gu, W. Yue, J. Hu, X. Niu, H. Tang et al., Asymmetrically coordinated Cu–N_1_C_2_ single-atom catalyst immobilized on Ti_3_C_2_T_x_ MXene as separator coating for lithium–sulfur batteries. Adv. Energy Mater. **13**(20), 2204014 (2023). 10.1002/aenm.202204014

[118] Y. Li, W. Wang, B. Zhang, L. Fu, M. Wan et al., Manipulating redox kinetics of sulfur species using Mott-Schottky electrocatalysts for advanced lithium-sulfur batteries. Nano Lett. **21**(15), 6656–6663 (2021). 10.1021/acs.nanolett.1c02161 34291943 10.1021/acs.nanolett.1c02161

[119] S. Deng, W. Sun, J. Tang, M. Jafarpour, F. Nüesch et al., Multifunctional SnO_2_ QDs/MXene heterostructures as laminar interlayers for improved polysulfide conversion and lithium plating behavior. Nano-Micro Lett. **16**(1), 229 (2024). 10.1007/s40820-024-01446-w 10.1007/s40820-024-01446-w PMC11213846 38940902

[120] L. Jiao, C. Zhang, C.N. Geng, S.C. Wu, H. Li et al., Capture and catalytic conversion of polysulfides by *in situ* built TiO_2_-MXene heterostructures for lithium–sulfur batteries. Adv. Energy Mater. **9**(19), 1900219 (2019). 10.1002/aenm.201900219

[121] C. Song, Q. Yan, T. Zhang, H. Lin, H. Ye et al., Enhanced polysulfide conversion through metal oxide-support interaction in MnO_*x*_/MXene. Chem. Eng. J. **420**, 130452 (2021). 10.1016/j.cej.2021.130452

[122] Y. Zhang, Z. Mu, C. Yang, Z. Xu, S. Zhang et al., Rational design of MXene/1T-2H MoS_2_-C nanohybrids for high-performance lithium–sulfur batteries. Adv. Funct. Mater. **28**(38), 1707578 (2018). 10.1002/adfm.201707578

[123] J.Z. Chen, Z.A. Li, J.T. Lei, P.P. Chen, D.L. Zhao, Accelerated ion-electron transport in bi-heterostructures constructed based on ohmic contacts for efficient bi-directional catalysis of lithium–sulfur batteries. Small **21**(2), 2408284 (2025). 10.1002/smll.202408284 10.1002/smll.202408284 39520321

[124] X. Wang, L. Chen, Y. Yu, W. Wang, L. Yue et al., Tuning p-band centers and interfacial built-In electric field of heterostructure catalysts to expedite bidirectional sulfur redox for high-performance Li–S batteries. Adv. Funct. Mater. **34**(41), 2406290 (2024). 10.1002/adfm.202406290

[125] M. Xu, Q. Zhu, Y. Li, Y. Gao, N. Sun et al., Atom-dominated relay catalysis of high-entropy MXene promotes cascade polysulfide conversion for lithium–sulfur batteries. Energy Environ. Sci. **17**(20), 7735–7748 (2024). 10.1039/D4EE03402C

[126] Y. Xu, W. Yuan, C. Geng, Z. Hu, Q. Li et al., High-entropy catalysis accelerating stepwise sulfur redox reactions for lithium–sulfur batteries. Adv. Sci. **11**(31), e2402497 (2024). 10.1002/advs.202402497 10.1002/advs.202402497 PMC11336958 38884340

[127] R. Wang, J. Jiao, D. Liu, Y. He, Y. Yang et al., High-entropy metal nitride embedded in concave porous carbon enabling polysulfide conversion in lithium–sulfur batteries. Small **20**(44), e2405148 (2024). 10.1002/smll.202405148 38978436 10.1002/smll.202405148

[128] J. Jiao, D. Liu, Y. He, Y. Shen, J. Zhou et al., Entropy engineering-modulated *d*-band center of transition metal nitrides for catalyzing polysulfide conversion in lithium–sulfur batteries. Small (2024). 10.1002/smll.202409740 10.1002/smll.202409740 39600081

[129] Q. Liang, S. Wang, X. Lu, X. Jia, J. Yang et al., High-entropy MXene as bifunctional mediator toward advanced Li-S full batteries. ACS Nano **18**(3), 2395–2408 (2024). 10.1021/acsnano.3c10731 38194614 10.1021/acsnano.3c10731

[130] Q. Zou, Q. Liang, H. Zhou, Y. Guo, J. Xue et al., Promoting Li_2_S nucleation/dissolution kinetics *via* multiple active sites over TiVCrMoC_3_T_*x*_ interface. Small **20**(40), e2402344 (2024). 10.1002/smll.202402344 38829023 10.1002/smll.202402344

[131] K. Wu, G. Lu, B. Huang, Z. Hu, Y. Lv et al., Entropy-driven highly chaotic MXene-based heterostructures as an efficient sulfur redox electrocatalysts for Li-S battery. Adv. Funct. Mater. **34**(45), 2404976 (2024). 10.1002/adfm.202404976

[132] X. Zuo, L. Wang, M. Zhen, T. You, D. Liu et al., Multifunctional TiN-MXene-Co@CNTs networks as sulfur/lithium host for high-areal-capacity lithium-sulfur batteries. Angew. Chem. Int. Ed. **63**(35), e202408026 (2024). 10.1002/anie.202408026 10.1002/anie.202408026 38867467

[133] B. Zhang, C. Luo, Y. Deng, Z. Huang, G. Zhou et al., Optimized catalytic WS_2_–WO_3_ heterostructure design for accelerated polysulfide conversion in lithium–sulfur batteries. Adv. Energy Mater. **10**(15), 2000091 (2020). 10.1002/aenm.202000091

[134] Y. Yan, H. Li, C. Cheng, T. Yan, W. Gao et al., Boosting polysulfide redox conversion of Li-S batteries by one-step-synthesized Co–Mo bimetallic nitride. J. Energy Chem. **61**, 336–346 (2021). 10.1016/j.jechem.2021.03.041

[135] W. Zhou, D. Zhao, Q. Wu, J. Dan, X. Zhu et al., Rational design of the *Lotus*-like N-Co_2_VO_4_-Co heterostructures with well-defined interfaces in suppressing the shuttle effect and dendrite growth in lithium–sulfur batteries. Small **17**(50), 2104109 (2021). 10.1002/smll.202104109 10.1002/smll.202104109 34708517

[136] R. Zhang, Y. Dong, M.A. Al-Tahan, Y. Zhang, R. Wei et al., Insights into the sandwich-like ultrathin Ni-doped MoS_2_/rGO hybrid as effective sulfur hosts with excellent adsorption and electrocatalysis effects for lithium-sulfur batteries. J. Energy Chem. **60**, 85–94 (2021). 10.1016/j.jechem.2021.01.004

[137] X. Huang, J. Tang, B. Luo, R. Knibbe, T.G. Lin et al., Sandwich-like ultrathin TiS_2_ nanosheets confined within N, S codoped porous carbon as an effective polysulfide promoter in lithium-sulfur batteries. Adv. Energy Mater. **9**, 1901872 (2019). 10.1002/aenm.201901872

[138] L. Shi, W. Yuan, J. Liu, W. Zhang, S. Hou et al., P-doped NiSe_2_ nanorods grown on activated carbon cloths for high-loading lithium-sulfur batteries. J. Alloys Compd. **875**, 160045 (2021). 10.1016/j.jallcom.2021.160045

[139] S. Li, P. Xu, M.K. Aslam, C. Chen, A. Rashid et al., Propelling polysulfide conversion for high-loading lithium–sulfur batteries through highly sulfiphilic NiCo_2_S_4_ nanotubes. Energy Storage Mater. **27**, 51–60 (2020). 10.1016/j.ensm.2020.01.017

[140] H. Gao, S. Ning, J. Zou, S. Men, Y. Zhou et al., The electrocatalytic activity of BaTiO_3_ nanoparticles towards polysulfides enables high-performance lithium–sulfur batteries. J. Energy Chem. **48**, 208–216 (2020). 10.1016/j.jechem.2020.01.028

